# Automated Functional Analysis of Astrocytes from Chronic Time-Lapse Calcium Imaging Data

**DOI:** 10.3389/fninf.2017.00048

**Published:** 2017-07-14

**Authors:** Yinxue Wang, Guilai Shi, David J. Miller, Yizhi Wang, Congchao Wang, Gerard Broussard, Yue Wang, Lin Tian, Guoqiang Yu

**Affiliations:** ^1^Bradley Department of Electrical and Computer Engineering, Virginia Polytechnic Institute and State University Arlington, VA, United States; ^2^Department of Biochemistry and Molecular Medicine, University of California Davis School of Medicine Davis, CA, United States; ^3^Department of Electrical Engineering, School of Electrical Engineering and Computer Science, Pennsylvania State University University Park, PA, United States

**Keywords:** astrocyte, astrocyte activity, functional phenotype, calcium dynamics, time-lapse calcium image, signal propagation

## Abstract

Recent discoveries that astrocytes exert proactive regulatory effects on neural information processing and that they are deeply involved in normal brain development and disease pathology have stimulated broad interest in understanding astrocyte functional roles in brain circuit. Measuring astrocyte functional status is now technically feasible, due to recent advances in modern microscopy and ultrasensitive cell-type specific genetically encoded Ca^2+^ indicators for chronic imaging. However, there is a big gap between the capability of generating large dataset via calcium imaging and the availability of sophisticated analytical tools for decoding the astrocyte function. Current practice is essentially manual, which not only limits analysis throughput but also risks introducing bias and missing important information latent in complex, dynamic big data. Here, we report a suite of computational tools, called Functional AStrocyte Phenotyping (FASP), for automatically quantifying the functional status of astrocytes. Considering the complex nature of Ca^2+^ signaling in astrocytes and low signal to noise ratio, FASP is designed with data-driven and probabilistic principles, to flexibly account for various patterns and to perform robustly with noisy data. In particular, FASP explicitly models signal propagation, which rules out the applicability of tools designed for other types of data. We demonstrate the effectiveness of FASP using extensive synthetic and real data sets. The findings by FASP were verified by manual inspection. FASP also detected signals that were missed by purely manual analysis but could be confirmed by more careful manual examination under the guidance of automatic analysis. All algorithms and the analysis pipeline are packaged into a plugin for Fiji (ImageJ), with the source code freely available online at https://github.com/VTcbil/FASP.

## Introduction

Astrocytes, which constitute nearly half the volume of the adult human brain, have long been considered to play only passive roles in the central nervous system, such as supplying trophic factors, maintaining ion homeostasis, and serving as an inert scaffold. In recent years, active roles in regulating various aspects of neuronal function have been identified (Agulhon et al., 2008; Khakh and Sofroniew, 2015; Bazargani and Attwell, 2016). Neuron-astrocyte communication at synapses regulates synaptic transmission and plasticity, breathing, memory formation, motor function, and sleep, and is implicated in many neuropsychiatric disorders (Haydon, 2001; Volterra and Meldolesi, 2005; Halassa and Haydon, 2010; Clarke and Barres, 2013). Astrocytes interact with synapses through release of soluble factors driven by intracellular Ca^2+^ elevations (Agulhon et al., 2008; Haustein et al., 2014) and, as a result, the Ca^2+^ dynamics are the best established correlates of the excitatory state and functional readout of astrocytes. With the convergence of recent advances in both modern microscopy and ultrasensitive cell-type specific genetic encoded calcium indicators (GECI; Knöpfel and Boyden, 2012; Broussard et al., 2014), it is now possible to conduct chronic optical imaging to record activities of a large number of astrocytes with high spatial and temporal resolution (Srinivasan et al., 2015), resulting in overwhelmingly large data sets and making manual analysis prohibitive. Note that the term “chronic imaging” used in this paper is essentially equivalent to “long-term imaging” which is also widely used in literature. However, effective automatic computational tools for analyzing functional astrocyte data have lagged far behind the capability of generating large-volume Ca^2+^ imaging data. Quantifying the functional status of astrocytes here relies on three core analytical tasks: (1) identifying astrocytic functionally independent units (FIUs), (2) estimating the characteristic curves of Ca^2+^ dynamics, and (3) extracting functional features of astrocytes or astrocytic FIUs. The detailed definition of FIU and characteristic curve is referred to Section Problem Statement and Formulation.

**Figure 1:**
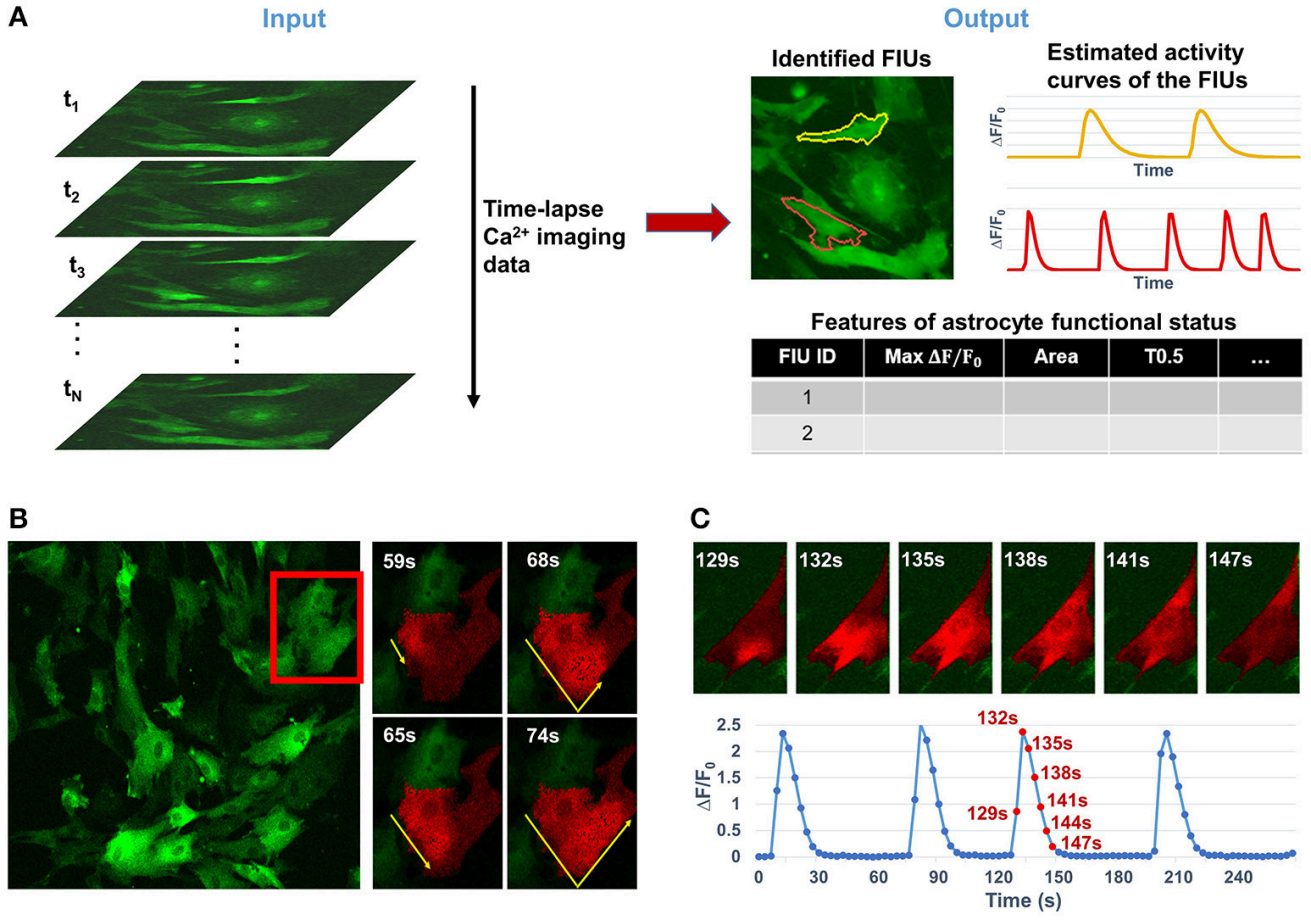
Overview of the analytical tasks and challenges in quantifying the functional dynamics of astrocytes from time-lapse astrocyte Ca^2+^ imaging data. **(A)** The problem in this study is to automatically quantify astrocyte functional dynamics from the input data, a time-lapse astrocyte Ca^2+^ image stack. It can be divided into three core analytical tasks: (1) identifying astrocytic functionally independent units (FIUs), (2) estimating the characteristic curves of FIUs' Ca^2+^ dynamics, and (3) extracting descriptive features of the astrocytic functional status. **(B)** A real example of astrocytes derived from human induced pluripotent stem cells (hiPSCs) shows the complex nature of astrocyte Ca^2+^ signaling which makes the computational analysis challenging. Astrocytic FIUs have heterogeneous and irregular morphological patterns; Ca^2+^ transients propagate within an astrocyte with complex speed and direction patterns; signal to noise ratio (SNR) is heterogeneous and varies in a wide range even in the same field of view. The red rectangle on the left image highlights the area shown on the right. **(C)** Illustrative example of slow propagation in another FIU, where the time lag between two ends of the FIU is in the same scale as the duration of a Ca^2+^ elevation event. This means we cannot eliminate effect of propagation by down-sampling, otherwise the signaling events will be removed, too.

Analyzing functional astrocyte data is challenging due to the complex nature of astrocyte Ca^2+^ signaling. First, one single astrocyte may contain multiple FIUs due to calcium compartmentalization, varying from large areas in soma to small and local microdomains in processes, which have different Ca^2+^ activity patterns. Thus, it is infeasible to use cellular information such as nucleus to identify FIUs. Second, Ca^2+^ elevations do not occur simultaneously in an FIU, but propagate as a Ca^2+^ wave within an astrocyte or between astrocytes with complex speed and direction patterns (Fiacco and McCarthy, 2006). Examples are shown in Figures 1B,C. The time delay between two parts in the same FIU can be larger than the duration of an event (Figure 1C; Fiacco and McCarthy, 2006), so it is impossible to eliminate time lags within an FIU by downsampling. As an intrinsic property of astrocyte Ca^2+^ signaling, signal propagation is quite prevalent (Fiacco and McCarthy, 2004, 2006; Matyash and Kettenmann, 2010). In our real data of *in vitro* human astrocyte induced pluripotent stem cells (hiPSCs), we found >90% astrocytic FIUs having discernable propagation. Thus, any modeling effort must explicitly take into account the propagation. As a similar but relatively better studied type of cellular excitation indicator, neuronal Ca^2+^ spikes also propagate, but the propagation is generally so fast that the signal can be regarded as synchronized, that is to say, any time lag between Ca^2+^ elevations in two parts of a cell is shorter than the temporal resolution of imaging. Consequently, the neuronal Ca^2+^ signal pattern can be considered as homogeneous in a cell from an image analysis point of view (Mukamel et al., 2009). Third, the morphological features of FIUs, such as size and shape, are irregular and heterogeneous, so morphology cannot be used for detection. Last but not least, heterogeneous expression of protein indicators and other factors give rise to a wide range of signal to noise ratios (SNRs) in the same field of view (Knöpfel and Boyden, 2012); and in a considerable proportion of FIUs, the Ca^2+^ signals have low SNR, due to the fundamental limitations of living Ca^2+^ imaging such as the existence of auto-fluorescence and imperfect selectivity of the calcium indicator (Myers, 2012). The low SNR demands sophisticated modeling to maximize information extraction and a rigorous statistical framework to accurately control the false positive rate. These challenges significantly increase the difficulty in repurposing existing methods designed for other dynamic imaging data, such as neuronal Ca^2+^ data, and necessitate development of new approaches.

Since complete astrocyte Ca^2+^ data is new, there are very few analytical tools specifically designed for astrocytes. The only two scripts to our knowledge are GECIquant (Srinivasan et al., 2015) and CaSCaDe (Agarwal et al., 2017). However, both scripts are “semi-automated,” involving significant manual effort which is prone to operator bias and variation. GECIquant requires manually setting multiple parameters, adjusting thresholds, and drawing polygons, while CaSCaDe needs manually labeling thousands of ROIs for training. In contrast, the large-scale data we are modeling requires a fully automated algorithm. In addition, to identify FIUs, GECIquant projects a raw image stack into a single map of pixel-wise temporally maximum intensity and then binarizes it using a user-given threshold, losing the rich information of temporal patterns and confounding active units with silent cells that have high baseline intensity. Similarly, CaSCaDe projects the image stack into a map by summing up all the intensities and uses a threshold to binarize the map. Most critically, even though both were designed for astrocyte data, signal propagation were neither modeled nor quantified.

More broadly, from a technical point of view, our problem falls under the general category of time-lapse Ca^2+^ image analysis. To date, a handful of algorithms have been developed for analyzing neuronal Ca^2+^ imaging data (Reidl et al., 2007; Mukamel et al., 2009; Smith and Häusser, 2010; Valmianski et al., 2010; Andilla and Hamprecht, 2013; Diego et al., 2013; Pachitariu et al., 2013; Kaifosh et al., 2014; Maruyama et al., 2014; Soelter et al., 2014; Pnevmatikakis et al., 2016). However, none of them can be applied to astrocytic Ca^2+^ data due to the specific challenges mentioned above. For more detailed discussion, see Section Necessity of Specific Tools for Analyzing Time-Lapse Astrocyte Ca^2+^ Imaging Data.

**Figure 2:**
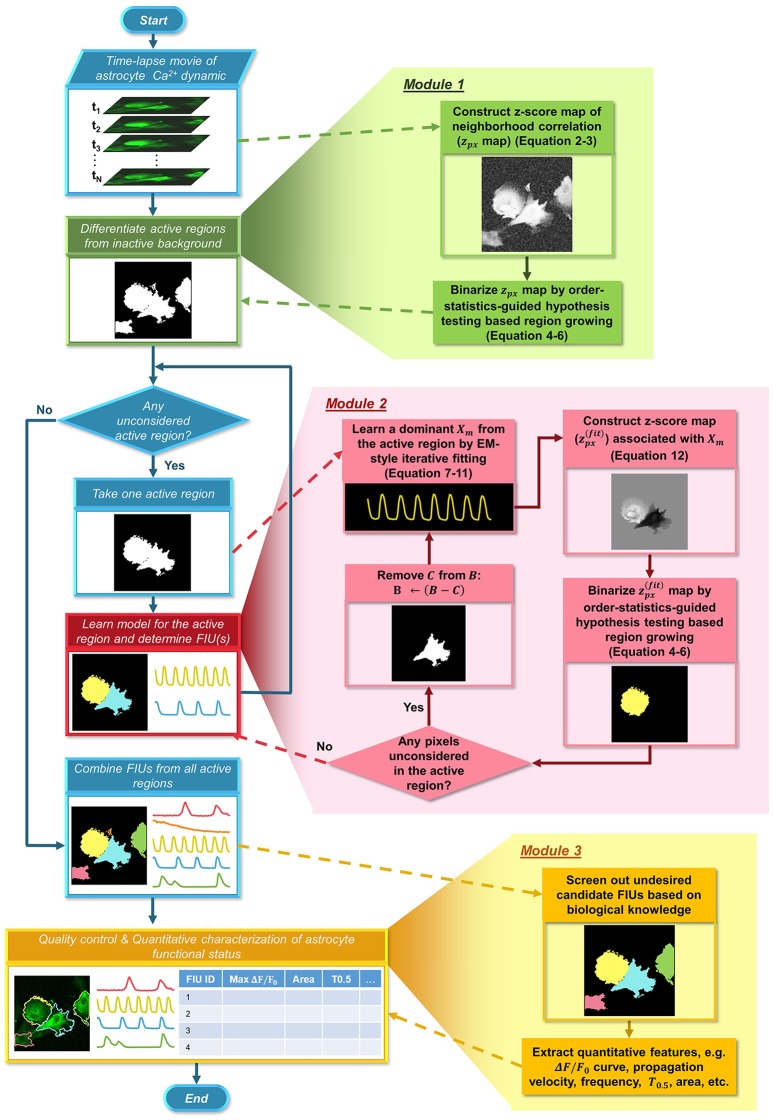
Flowchart of the proposed FASP algorithm. FASP addresses the problem using three modules. The blue pipeline on the left shows the major structure of FASP, including the relationship among the three modules. More detailed procedures are depicted in the expanded blocks of the modules. The output of each step or procedure is given right under the procedure description.

Here, we formulate a statistical model for astrocyte Ca^2+^ dynamics imaging data, and propose an integrated suite of algorithms, Functional AStrocyte Phenotyping (FASP), to simultaneously identify astrocytic FIUs, extract their functional features, and further characterize the functional status. The flowchart of FASP is shown in Figure 2. FASP possesses several unique features. First, recognizing that the core tasks—the identification of FIUs, the extraction of the corresponding characteristic curves and the estimation of propagation patterns—are mutually dependent, FASP addresses them in a unified way by building an integrated probabilistic model. Second, FASP is data-driven, learning model parameters using machine learning techniques without constraints on the form of the characteristic signals, the morphological patterns of FIUs, the spatial distribution/sparsity of FIUs, or the total number of FIUs. Thus, it can flexibly account for various Ca^2+^ events including waves and microdomain fluctuations, heterogeneous morphology, a large range of unit sizes and spatial intensities of FIUs, and, critically, it does not require a pre-assumed total number of FIUs. Third, FASP models Ca^2+^ propagation explicitly, dealing with cases with or without intracellular propagation phenomena. This explicit modeling not only contributes to faithfully identify FIU, but also enables extracting characteristic features related to propagation patterns. Fourth, FASP takes full advantage of spatial structural information to facilitate learning and enhance performance. Lastly but very importantly, it is deeply probabilistically principled. By judicious application of various statistical theories, FASP accurately distinguishes signals from noises and controls false positive rate, which is essential for analyzing noisy data. FASP also confers tuning parameters with probabilistic meaning, greatly facilitating usability of parameter setting.

We evaluated FASP on both synthetic data simulating Ca^2+^ signaling and real data of *in vitro* human astrocyte induced pluripotent stem cells (hiPSCs). The quantitative evaluation demonstrated FASP's effectiveness, flexibility, and robustness. Compared to a manual ROI drawing, FASP generated more accurate contour and detected quite a few otherwise missed activities. The experimental comparison between FASP and a representative of neuron-targeted methods that assume in-FIU synchronization (Mukamel et al., 2009) validated the necessity of developing methods specifically modeling intracellular propagation, and also showed the superior performance of FASP for this task. To further validate our method, we applied FASP to study agonist-induced Ca^2+^ activities in *in vitro* rat astrocytes. FASP successfully detected the induced FIUs responding to three known agonists for astrotytic Ca^2+^ signaling: ATP, glutamate and 3,5-dihydroxyphenylglycine (3,5-DHPG). Many quantitative features and patterns of the astrocytic activities revealed by FASP are consistent with what have been reported in the literature.

## Methods

### Problem statement and formulation

For convenience of discussion, we consider time-lapse 2D imaging data, which can be easily extended to the 3D imaging case. We formulate the astrocyte Ca^2+^ data as a 3-dimensional array *Y*[*i, j, t*], where *i* ∈ {1, 2, …, *D*_*h*_} is the horizontal spatial index, *j* ∈ {1, 2, …, *D*_*v*_} is the vertical spatial index, and *t* ∈ {1, 2, …, *D*_*t*_} the temporal index. Define an astrocytic FIU as a group of spatially connected pixels sharing a characteristic Ca^2+^ temporal pattern with possibly different temporal phases and noises. Define the characteristic curve of an astrocytic FIU as the core time series of fluorescence intensity *F* shared by pixels in the FIU, from which the Ca^2+^ dynamics quantified as Δ*F*/*F*_0_ = (*F* − *F*_0_)/*F*_0_ can be obtained, where *F*_0_ is the baseline fluorescence. We model the fluorescence microscopy imaging data of astrocyte Ca^2+^ dynamics *Y*[*i, j, t*] consisting of *M* FIUs as

(1) $$\begin{array}{l} Y\left[i,j,t\right]=\beta_{0}\left[i,j\right]+\sum_{m=1}^{M}\beta_{m}\left[i,j\right]X_{m}\left[t-\tau_{ij}\right]+\varepsilon [i,j,t],\\ s.\;t.\left\{ \begin{array}{l} {\left\|X_{m}\right\|}_{2}=1,E\left[X_{m}\right]=0,\text{ for }m=1,\;2,\ldots ,M\\ \;\sum_{m=1}^{M}I\left(\beta_{m}\left[i,j\right]\neq 0\right)\leq 1,\text{ for }i=1,2,\ldots ,D_{h},\\ \;j=1,2,\ldots ,D_{v}\\ \;c\left(G\left(\beta_{m}\right)\right)\leq 1,\text{ for }m=1,2,\ldots ,M \end{array}\right. \end{array}$$

where β_0_[*i, j*] is a constant associated with each pixel [*i, j*]; β_*m*_[*i, j*] is the coefficient for the *m*th FIU; *X*_*m*_ is the characteristic curve of the *m*th FIU; τ_*ij*_ is the time lag of [*i, j*]'s time-intensity curve with respect to the characteristic curve, as a result of calcium signal propagation; and ε [*i, j, t*] is an independent Gaussian noise which, given any pixel [*i, j*], has zero mean and a common variance $\sigma_{0}^{2}\left[i,j\right]$ across different time points *t*. Note that image noise is often non-Gaussian with intensity-dependent variance, but we can apply variance stabilization technique to the data to satisfy the assumption of Gaussian noise with common variance (Starck et al., 1998; Foi et al., 2008). The constraint ||*X*_*m*_||_2_ = 1 removes the model non-identifiability arising from multiplying *X*_*m*_ by a factor and dividing β_*m*_ by the same factor. *I*(·) is an indicator function and the constraint $\overset{M}{\underset{m=1}{\sum }}I\left(\beta_{m}\left[i,j\right]\neq 0\right)\leq 1$ ensures that each pixel will be associated with at most one FIU. *c*(*G*(β_*m*_)) is the number of connected components in the graph induced by the non-zero β_*m*_[*i, j*] with the edges derived from the pixel neighborhood structure. The constraint *c*(*G*(β_*m*_)) ≤ 1 ensures all pixels associated with the same FIU are connected. Model (1) assumes no cell migration or deformation, or that the data is preprocessed by migrating cell tracking and registration techniques.

We primarily concentrate upon three core tasks in analyzing astrocyte Ca^2+^ data, especially the first two: (1) the identification of FIUs and (2) the estimation of the corresponding characteristic curves; and (3) the extraction of biologically interesting quantitative features of astrocyte functional status. Based on model (1), the first two tasks can be addressed by learning the parameters β_*m*_[*i, j*] and *X*_*m*_[*t*], for *m* = 1, 2…, *M*. The *m*th FIU is the collection of all pixels with non-zero β_*m*_; the corresponding characteristic curve can be obtained directly from *X*_*m*_. Hence, in this report we focus on the model learning. Note that automatically determining the number of FIUs, *M*, is an important part of learning, and that accurate estimation of $\tau_{ij}^{\prime }s$ is necessary for the correct estimation of *X*_*m*_. Put simply, the problem is to learn all parameters, β_0_[*i, j*], β_*m*_[*i, j*], *X*_*m*_[t], τ_*ij*_ and *M* from the observed data *Y*[*i, j, t*]. This is a mathematically challenging problem, because the number of parameters is huge and all parameters interact in a highly non-linear way to give rise to the observed data. In the following, we will discuss how a bunch of advanced probabilistic and machine learning techniques are integrated as FASP to solve the problem.

### Overview and design principles of FASP

Our method, FASP, addresses the problem using three major modules (Figure 2). First, it distinguishes “active regions” from inactive background. In this paper, we call a pixel an “active pixel” if it has a non-zero β_*m*_[*i, j*] for some *m* in model (1), and call a region an “active region” if it contains only active pixels and all its neighboring pixels are “inactive,” or, not active pixels. Second, given each active region, FASP sequentially identifies distinct FIUs within the active region one by one and estimates the corresponding characteristic curves. It also automatically determines the number of FIUs. Finally, it performs quality control by automatic post processing and proofreading, and computes various quantitative features to characterize the functional status of astrocytes.

Learning model (1), or jointly estimating β_*m*_, *X*_*m*_, and τ_*ij*_ with multiple indices *m*, on the entire field of view simultaneously will be very time-consuming and may be prone to severe overfitting. As a critical part to make the whole strategy feasible, we designed two schemes to combat this problem. Firstly, we designed a special map of neighborhood correlation so that FASP can, in its first module, detect active regions, without needing to explicitly specify the unknown *m* for any pixel. As outputs of the first module, we obtain a set of active regions. Secondly, in a given active region, different pixels may have non-zero β_*m*_[*i, j*] for different *m*: multiple FIUs may be spatially connected and hence pixels of them may be in the same active region, which constantly occurs in astrocyte Ca^2+^ data. So we resort to a sequential approach to identify the FIUs in the same region one by one. In this multi-step framework, by constraining the model learning problem to active regions only and using sequential identification, the parameter search space becomes much smaller than the original space. In this way, FASP successfully mitigates the computational burden and relieves the potential overfitting problem.

In addition, there are other two critical principles for modeling astrocytic FIUs. First, fully utilizing spatial neighborhood information could effectively assist the model learning. According to the definition of FIUs, the spatial distribution of an FIU's pixels is localized, that is, pixels belonging to the same FIU must be spatially connected. Besides, neighboring pixels in an FIU should be similar in terms of signal pattern. We expect that incorporating neighborhood information will lead to higher statistical power to detect active regions and FIUs, compared with inspecting each pixel individually. Second, due to the nature of Ca^2+^ imaging, large noises (small signal-to-noise ratio) are typically observed and challenging the performance of analysis. In such cases, probabilistic principles are needed to enable information extraction under great uncertainty. We embrace the probabilistic principles in all steps of algorithm development. A set of statistical tools are exploited—maximum likelihood estimation for characteristic curve learning, Bayesian decision theory for distinguishing adjacent but different FIUs, Fisher's transformation and order statistics theory for hypotheses testing and error control. This principle is also crucial for practical use, as it allows end users to determine the number of FIUs and to anticipate the false positive rate. The statistical framework also helps to endow parameters with either physical or probabilistic meaning, which makes parameter tuning more objective.

### Module 1: detecting active regions

In order to constrain the parameter search space and thus facilitate the model learning, we first detect active regions. From a different point of view, in this module, FASP identifies all background or inactive pixels, and estimates their β_*m*_s to be zero for all *m*, with β_*m*_s of active pixels and *X*_*m*_s kept unknown. This is feasible owing to the fact that any two active pixels sharing the same characteristic curve *X*_*m*_ are expected to have higher correlation than those which do not, so the correlation between neighboring pixels alone is sufficient for inferring active regions. Therefore, in this module, we first build a map of neighborhood correlation in which the value at each pixel is basically the correlation between this pixel's intensity time series with its neighboring pixels'. The formulation of neighborhood correlation score is given in Section Building a Map of Correlation between Neighboring Pixels. Then the problem of detecting active regions is translated into a problem of binarizing this neighborhood correlation map, distinguishing true correlation between signals from random correlation due to noises.

In applications with low-SNR, probabilistic models and statistical approaches often play positive roles in suppressing the impact of large noises. Besides, significance assessment is often critically important for biomedical applications, as we care much about false positives, but no existing image segmentation method to our knowledge gives significance assessment. Thus, we aim to build a probabilistically principled strategy for detecting active regions, or, segmenting the neighborhood correlation map. To this end, we construct a z-score map from the correlation map (see Section Constructing a Z-Score Map of Neighborhood Correlation), endowing the correlation scores explicit probabilistic meaning. Any background pixel should have a correlation z-score following a standard Gaussian distribution (the null distribution), while active pixels are expected to have statistically significantly large positive z-score. This transformation also stabilizes the variance, which means to make the variance of score independent from its magnitude, assuring that all pixels will be treated equally in the following analysis.

Having got the correlation z-score map, rather than testing each pixel in isolation (e.g., do thresholding to the z-score), FASP evaluates a set of connected pixels as a whole so that the cumulative information in spatial neighborhoods is fully utilized to enhance confidence in decisions. We develop a region growing algorithm, starting from a singleton containing only one pixel, known as “seed,” and iteratively growing the region, or pixel set, until it reaches the greatest statistical significance. These procedures are repeatedly conducted till all qualified seed pixel singletons are labeled as searched. The main difficulty and a key technique here lies in how to assess the statistical significance of a region, since the operations that FASP does to enlarge the region in each iteration introduce some dependency structure among the pixels. Realizing that such a structure can be modeled using order statistics, we design a region-level hypothesis test based on the theory of order statistics, which is one of the major innovations of our work. We will introduce this region growing method in detail in Section Active Region Detection on Z-Score Map by Order-Statistics Guided Hypothesis Testing, and compare it with three other popular segmentation methods in Section The Binarization Method Based on Order-Statistics Theory is Effective.

#### Building a map of correlation between neighboring pixels

**Figure 3:**
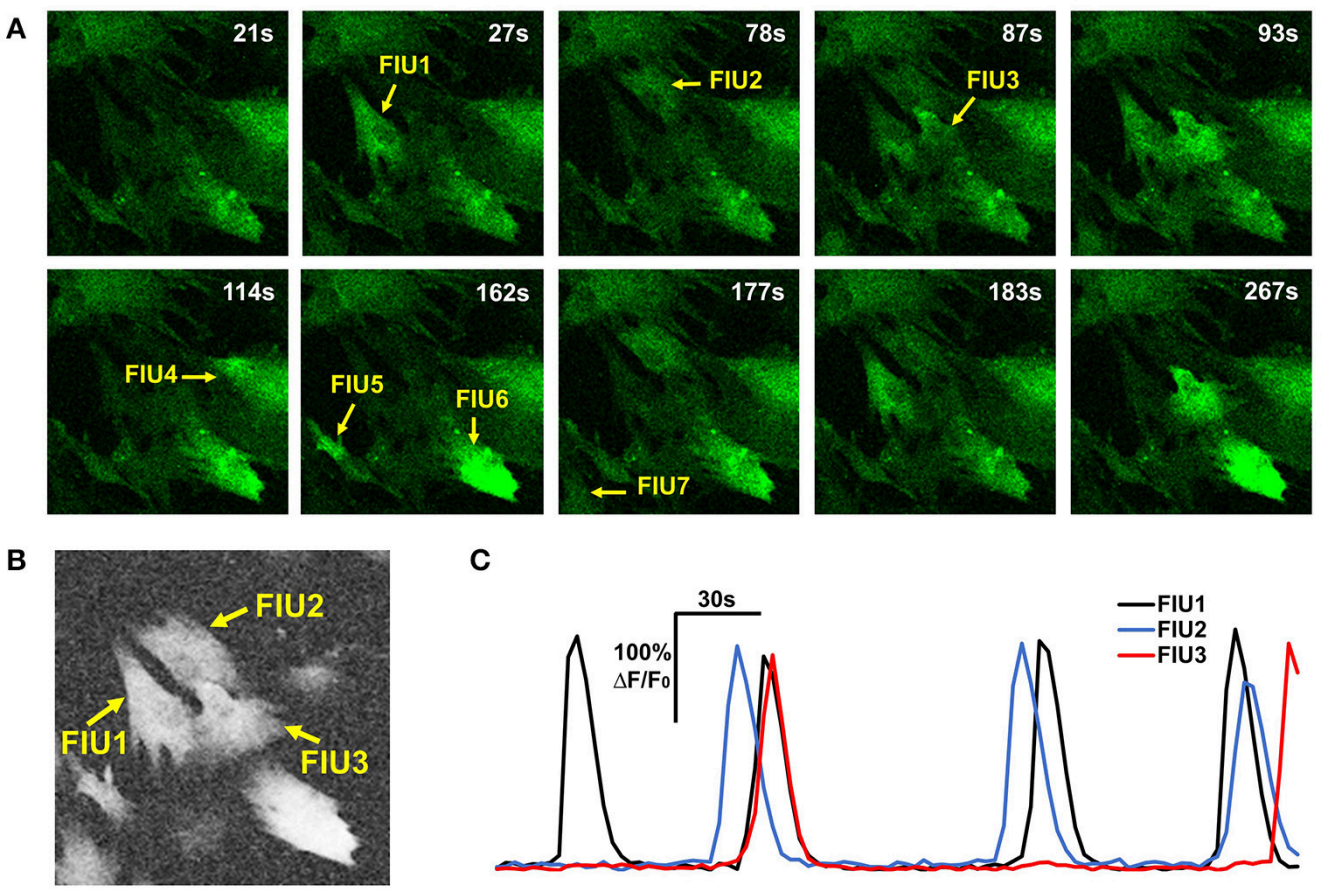
Properties of astrocyte time-lapse Ca^2+^ imaging data that served as inspirations or constraints in the development of FASP. **(A)** A time-lapse imaging data sample of hiPSCs-derived astrocytes contains 7 FIUs with different Ca^2+^ dynamics, among which FIU1, 2, and 3 are spatially connected. **(B)** The neighborhood correlation (*r*_*sc*_) map of the sample shown in **(A)**. Pixels within FIUs shows obviously higher neighborhood correlation than those in the non-FIU regions. **(C)** The characteristic curves of FIU1, 2, and 3 in **(A)**. With slight time shifts, each pair of curves among the three have coincident peaks, suggesting non-zero correlation between the curves.

We define the neighborhood correlation at pixel [*i, j*] as

(2) $$r_{sc}[i,j]=cor\left(Y[i,j,:],{\bar{Y}}_{nb}[i,j,:]\right)$$

where *cor*(,) is the Pearson correlation coefficient between two curves; ${\bar{Y}}_{nb}\left[i,j,:\right]$ is the average curve of the eight direct-neighbor pixels of [*i, j*]. Inspection of real data confirmed that the neighborhood correlation at pixel [*i, j*] indicates how active this pixel is. For example, comparing Figures 3A,B, we can see that pixels in FIUs have much higher neighborhood correlation than the pixels out of FIUs.

One may notice that we intentionally ignore the time shifts between neighboring pixels. Although, for two arbitrary pixels in the same FIU the time shifts can be quite different due to propagation, we expect that the time shifts between neighboring pixels are minor and can be ignored in computing the average curve and the correlation coefficient. For cases where SNR is high while propagations are very slow (time shifts between neighboring pixels are large), we also designed an alternative neighborhood correlation score to explicitly model time shifts and thus better deal with the Ca^2+^ propagation phenomena, with a price of losing some power to suppress noises. More discussion and comparison of the two definitions are given in Section Alternative Design of Neighborhood Correlation Score.

#### Constructing a Z-score map of neighborhood correlation

We make a monotonic transformation to each pixel in the neighborhood correlation map:

(3) $$\begin{array}{c} z_{px}[i,j]=F(r_{sc}[i,j])=\frac{\sqrt{N-3}}{2}\ln\left(\frac{1+r_{sc}[i,j]}{1-r_{sc}[i,j]}\right), \end{array}$$

where *F*(·) denotes the normalized Fisher transformation (Fisher, 1915, 1921), and *N* is the total number of time points.

According to the properties of the Fisher transformation, *z*_*px*_[*i, j*] asymptotically follows a standard Gaussian distribution under the null hypothesis that the pixel [*i, j*] has zero β_*m*_ for all *m*, that is, [*i, j*] is not associated with any FIU. If the pixel [*i, j*] belongs to one FIU, the time curve at the pixel [*i, j*] will be correlated with the ones at its neighboring pixels because of the spatial localization property of FIU. As a consequence, *r*_*sc*_[*i, j*] is expected to be positive. Due to the monotonicity of the normalized Fisher transformation, *z*_*px*_[*i, j*] is thus expected to be larger than 0. Note that the normalized Fisher transformed score always follows Gaussian distributions with constant variance of 1, regardless of the expected value of the score, or in other words, no matter in the background or in FIUs. The chosen transformation facilitates subsequent probabilistic analysis owing to its effect of variance stabilization and the good properties of Gaussian distributions.

#### Active region detection on Z-score map by order-statistics guided hypothesis testing

Having obtained a score map *z*_*px*_ computed as in Equation (3), FASP binarizes the map into foreground (active regions) and background (inactive regions) using a region growing strategy. Each round of region growing process starts from a singleton{*a*_0_}, where the seed pixel *a*_0_ has the largest *z*_*px*_ among the remaining pixels that have not been labeled as “searched.” Then the region (pixel set) iteratively grows. In each iteration, from the set of directly neighboring pixels of the current region we select a subset and attach it to this region, such that the resulting new region's statistical significance (given by a hypothesis test defined in the following paragraphs) is increased from the current region as much as possible. The growing stops if the termination criterion is reached, that is, the region's statistical significance no longer increases as this region continues to grow. It can be expected that, being expanded from a seed pixel, the most statistically significant region does not contain silent background pixels, because inclusion of silent pixels moves the test statistics toward the null. Once a round of region growing process is finished, another round is started as long as any pixel remained not labeled as “searched.” Finally the union of all found regions forms the map of active regions.

Both the selection of neighboring pixels and the termination criterion rely on the assessment of statistical significance of regions. Given a set of pixels, the null hypothesis here states that all pixels in this set are inactive pixels, in other words, their *z*_*px*_s all approximately follow a standard Gaussian distribution. Thus, the alternative hypothesis is that some pixels in this region are active pixels and their *z*_*px*_s tend to be positive. Note that a pixel set containing both active and inactive pixels is expected to have lower significance than its subsets containing active pixels only. As a consequence, the procedure of always finding the maximum significance rules out such cases.

Under the null hypothesis, the pixel-level z-scores *z*_*px*_ of all pixels in a region can be considered as independently standard-Gaussian distributed. However, the average *z*_*px*_ in each candidate region in the region growing process does not follow the Gaussian distribution suggested by the central limit theorem, because these pixels are conditioned on the operation of region growing and thus not independent anymore. The conditioning causes biases in both mean and variance of the average *z*_*px*_. Indeed, if these pixels are still approximately treated as independent standard Gaussian noises under the null hypothesis, the significance assessment can be quite straightforward using the central limit theorem. For comparison, we also experimented with the same region growing procedure but assessed significance using the central limit theorem, but it resulted in an endlessly expanding web of thin strings in simulation studies when no active regions were embedded.

Hence, a more sophisticated approach is needed to assess the statistical significance for each candidate active region. One critical innovation in the proposed framework is the realization that the biases and dependency structure come from the ranking and selection operation in the region growing procedure and the significance can thus be modeled by order-statistics theory (David and Nagaraja, 2003). Denote the current region *A* = {*a*_1_, *a*_2_, …, *a*_*n*_*A*__} and the surrounding set of pixels *B* = {*b*_1_, …, *b*_*n*_*B*__}. A candidate region *C* has the form *C* = *A* ∪ *B*′, where *B*′ is a subset of *B*. We have a score for the region *C* = {*c*_1_, *c*_2_, …, *c*_*k*_, …, *c*_*n*_*C*__} as,

(4) $$s(C)=\frac{1}{\sqrt{n_{C}}}{\sum^{\text{​}}}_{k=1}^{n_{C}}z_{px}[c_{k}]$$

where *z*_*px*_[*c*_*k*_] is the z-score of neighborhood correlation of pixel *c*_*k*_. Let us sort increasingly all pixels from *A* ∪ *B* by their *z*_*px*_ scores and record the ascending orders of elements of *C* as *J*. Thus, *J*[*k*] denotes the ascending order of pixel *c*_*k*_ in *A* ∪ *B* regarding *z*_*px*_. Based on the asymptotic distribution of linear combination of order statistics, known in the order-statistics theory (David and Nagaraja, 2003), the score *s*(*C*) can be approximated by a normal distribution with the following mean *E*[*S*(*C*)] and variance *Var*(*S*(*C*)),

(5) $$\begin{array}{c} E[S(C)]=\frac{1}{\sqrt{n_{C}}}{\sum^{\text{​}}}_{k=1}^{n_{C}}\Phi^{-1}(v_{k}), \end{array}$$

(6) $$\begin{array}{l} \begin{array}{c} Var(S(C))=\frac{2}{n_{C}(n_{A}+n_{B})}{\sum^{\text{​}}}_{k_{1}=1}^{n_{C}}{\sum^{\text{​}}}_{k_{2}=k_{1}}^{n_{C}} \end{array}\\ \;\begin{array}{c} \frac{v_{k_{2}}(1-v_{k_{1}})}{\phi (\Phi^{-1}(v_{k_{1}}))\phi (\Phi^{-1}(v_{k_{2}}))}, \end{array} \end{array}$$

where *v*_*k*_ = (*J* [*k*] + 0.5)/(*n*_*A*_ + *n*_*B*_), ϕ(·) is the standard Gaussian PDF and Φ^−1^(·) is the standard inverse Gaussian CDF. The essential idea behind (5) and (6) is that a linear combination of ordered variables is asymptotically normally distributed as the number of variables increases.

### Module 2: identifying FIUs and extracting characteristic curves

As shown in Figure 3 and Supplementary Video 1, it was repeatedly observed that spatially-connected FIUs can have different signal patterns, indicating that one active region can contain multiple FIUs. Identifying FIUs from an active region requires estimating all *X*_*m*_s that have non-zero β_*m*_s in this active region and assigning each pixel to the most likely *X*_*m*_. Learning all FIUs' parameters in parallel without knowing the number of FIUs in this active region is very time consuming, algorithmically complex, and prone to overfitting. A feasible alternative way is to resort to a sequential approach: in each round we seek for one *X*_*m*_ with non-zero β_*m*_ at some pixels in the active region, and then determine the spatial area of the *m*th FIU by finding pixels whose curves are well-explained by *X*_*m*_. Once an FIU is detected, we inspect its statistical significance: if its *p*-value is smaller than a given threshold, all pixels in this FIU are removed from the active region, and in the remaining region FASP continues to search for more FIUs until no significant FIU can be found. It is worth noting that, through this sequential strategy, the number of FIUs is determined by setting threshold for the statistical significance, which is easy and intuitive. We leave the significance threshold as a parameter for users to specify. Consequently, users can control the number of FIUs by setting different significance thresholds for claiming a significant FIU.

Thus, the three main components in this module respectively aim to: (1) learn an *X*_*m*_ and its corresponding parameters β_*m*_, τ, and $\sigma_{0}^{2}$ at each pixel in the remaining part of the active region (Section Learning Model Parameters β_*m*_, *X*_*m*_, τ, and σ^2^ Associated with the *m*th FIU); (2) create a z-score map assessing how each pixel is likely to belong to the *m*th FIU (Section Constructing a Pixel-level Z-Score Map Associated with the *m*th FIU); (3) identify the *m*th FIU's pixel set on the z-score map. The third task can be accomplished by re-applying the order statistics guided region growing strategy as discussed previously in Section Active Region Detection on Z-Score Map by Order-Statistics Guided Hypothesis Testing, so here we focus on the former two in this module.

#### Learning model parameters β_m_, x_m_, τ, and σ^2^ associated with the *m*^th^ FIU

FASP employs a sequential strategy to alleviate the burden and drawbacks of simultaneously estimating all FIUs' parameters. However, even within one round of sequential model learning, where we only deal with one FIU, model learning is still a non-trivial problem because the parameters, *X*_*m*_, β_*m*_, τ, and $\sigma_{0}^{2}$, are all unknown and we have to estimate them at the same time. To tackle this problem, we developed an iterative, alternating approach reminiscent of the expectation-maximization (EM) algorithm (Dempster et al., 1977; Little and Rubin, 2014) to learn the model parameters. An *X*_*m*_ is initialized as the time-intensity curve of the pixel with the largest *z*_*px*_ in the region. Denote this initial pixel as [*i*_0_, *j*_0_]. τ[*i*_0_, *j*_0_] is initialized as 0. In each iteration, given a current estimate of *X*_*m*_, for each pixel [*i, j*] we update the other parameters as follows:

(7) $$\begin{array}{l} \begin{array}{c} {\hat{\tau }}^{*}[i,j]=\hat{\tau }[i_{nb},j_{nb}]+{\text{argmax}}_{\Delta t\in [U_{\tau },U_{\tau }]} \end{array}\\ \;\begin{array}{c} \left\{cor\left(Y[i,j,:+\hat{\tau }[i_{nb},j_{nb}]+t],\;{\hat{X}}_{m}\right)\right\} \end{array} \end{array}$$

(8) $${\hat{\beta }}_{m}^{*}\begin{array}{c} \left[i,j\right]={\hat{X}}_{m}^{T}\;Y\left[i,j,:+{\hat{\tau }}^{*}[i,j]\right] \end{array}$$

(9) $${\hat{\sigma }}_{0}^{2*}\begin{array}{c} \left[i,j\right]=\frac{1}{T}{\sum^{\text{​}}}_{t=1}^{T}{\left(Y\left[i,j,t+{\hat{\tau }}^{*}[i,j]\right]-{\hat{\beta }}_{m}^{*}[i,j]{\hat{X}}_{m}\right)}^{2} \end{array}$$

where *Y*[*i, j*, : + τ [*i, j*]] means the curve *Y*[*i, j*, :] shifted by τ[*i, j*]; $\hat{\tau }\left[i_{nb},j_{nb}\right]$ is the estimated time shift of pixel [*i, j*]'s neighbor [*i*_*nb*_, *j*_*nb*_]. In Equation (7), FASP basically searches for the best τ[*i, j*] that gives the highest correlation between *Y*[*i, j*, : + τ[*i, j*]] and ${\hat{X}}_{m}$. Thanks to the spatial continuity of Ca^2+^ propagation, the candidate range of $\hat{\tau }\left[i,j\right]$ can be narrowed to a small window of size 2*U*_τ_ around the time shift of the neighbor [*i*_*nb*_, *j*_*nb*_], thus we actually search for the best Δ*t* ∈ [−*U*_τ_, *U*_τ_] that leads to the highest correlation between $Y\left[i,j,:+\hat{\tau }\left[i_{nb},j_{nb}\right]+\Delta t\right]$ and ${\hat{X}}_{m}$. After scanning all pixels in the region and having their ${\hat{\beta }}_{m},\hat{\tau }$ and ${\hat{\sigma }}_{0}^{2}$ all updated to ${\hat{\beta }}_{m}^{*},\;{\hat{\tau }}^{*}$ and ${\hat{\sigma }}_{0}^{2*}$, we update *X*_*m*_ as a weighted summary of all *n* pixels within the region:

(10) $${{\hat{X}}^{\prime }}_{m}\begin{array}{c} ={\sum^{\text{​}}}_{k=1}^{n}\frac{{\hat{\beta }}_{m}^{*}[i_{k},j_{k}]}{{\hat{\sigma }}_{0}^{2*}[i_{k},j_{k}]}Y\left[i_{k},j_{k},:+\;{\hat{\tau }}^{*}[i_{k},j_{k}]\right] \end{array}$$

(11) $$\begin{array}{c} {\hat{X}}_{m}^{*}=\frac{{{\hat{X}}^{\prime }}_{m}}{||{{\hat{X}}^{\prime }}_{m}|{|}_{2}}. \end{array}$$

The parameter updating is iterated until convergence is reached. Here we decide the convergence when the relative change of ${\hat{X}}_{m}$ between two iterations, defined as $sd\left({\;\hat{X}}_{m}^{*}-{\hat{X}}_{m}\right)/sd\left({\;\hat{X}}_{m}^{*}\right)$, is less than a given threshold (*sd*(·) means the standard deviation over time points,${\;\hat{X}}_{m}^{*}$ is the estimate in current iteration, and ${\hat{X}}_{m}$ is the estimate in last iteration). The learning algorithm proposed here is experimentally observed to converge robustly. Indeed, in our experiments, we have found that the updating often converges in a dozen or fewer iterations.

#### Constructing a pixel-level z-score map associated with the*m*^th^ FIU

Given the estimated parameters associated with the *m*^*th*^ FIU, we construct a z-score map within the active region under inspection, where the z-score value at any active pixel indicates how likely this pixel belongs to the *m*^*th*^ FIU rather than other FIUs. The goal is to design a feature (score) differentially distributed at pixels in FIU *m* and at pixels of other FIUs. We expect that pixels in FIU *m* should have activities that are highly correlated with *X*_*m*_. Hence, this correlation, or equivalently the value of β_*m*_, can be used to determine which pixels belong to FIU *m*. However, the characteristic curves from different FIUs are often not mathematically orthogonal to each other, suggested by observations from real data (Figure 3C). Consequently, the curve of a pixel belonging to another currently unknown FIU *m*′ (*m*′ ≠ *m*), which is characterized by $X_{m^{\prime }}$, can also be correlated with *X*_*m*_ due to moderate but non-negligible correlation between *X*_*m*_ and $X_{m^{\prime }}$. In such a case, by simply assessing goodness of fit to *X*_*m*_, it is difficult to well-distinguish pixels in FIU *m* from pixels in FIU *m*′. In Section Neighborhood Correlation in Residual Signals Enables a Sequential FIU Identification, we have more detailed discussion on this problem. To solve it, here we propose a method to evaluate the confounding effects of other (latent) FIUs without knowing their characteristic curves, which is another important innovation in our work. By combining the measure of goodness of fit to *X*_*m*_ and this measure of confounding effects of other FIUs, FASP can well tell apart FIU *m* and others.

From the model learning procedure proposed in Section Learning Model Parameters β_*m*_, *X*_*m*_, τ, and σ^2^ Associated with the *m*^*th*^ FIU, we have obtained ${\hat{X}}_{m}$ and corresponding ${\hat{\beta }}_{m},\;\hat{\tau }$ at each pixel. We realized that, if a pixel is actually in FIU *m*′(*m* ≠ *m*′), the residual $\left(Y\left[t\right]-{\hat{\beta }}_{0}-{\hat{\beta }}_{m}{\hat{X}}_{m}\left[t-\hat{\tau }\right]\right)$ contains not only noise ε but also some true signal that cannot be explained by *X*_*m*_ but can be explained by $X_{m^{\prime }}$. We further realized that residuals of any two pixels in the same FIU *m*′ (*m*′ ≠ *m*) should be correlated as a result of their common correlation to $X_{m^{\prime }}$, while residuals of pixels in FIU *m* are independent. This key observation directly leads to the conclusion that the neighborhood correlation in residuals can be used as a measure of confounding effects of other FIUs. More discussion can be found in Section Neighborhood Correlation in Residual Signals Enables a Sequential FIU Identification. Accordingly we designed a special pixel-level score, inspired by Bayesian decision theory and indicating the competition between FIU *m* and other FIUs, as follows:

(12) $$\begin{array}{l} \begin{array}{c} z_{px}^{\left(fit\right)}[i,j]=\frac{1}{\sqrt{2}}\Phi^{-1}(\Phi^{2U_{\tau }}(F(r_{fit}[i,j])))- \end{array}\\ \;\begin{array}{c} \frac{1}{\sqrt{2}}F(r_{res}[i,j]) \end{array} \end{array}$$

where *r*_*fit*_[*i, j*] is the correlation coefficient between pixel [*i, j*]'s observed curve and the characteristic curve *X*_*m*_; *r*_*res*_[*i, j*] is the correlation coefficient between pixel [*i, j*]'s residual signal and the average residual signal of its eight direct neighboring pixels; Φ(·) and Φ^−1^(·) respectively represent the standard Gaussian CDF and its inverse; *F*(·) denotes the normalized Fisher transformation [the same as in Equation (3); (Fisher, 1915, 1921)]; and *U*_τ_ > 0 is the half window size of the candidate pool of τ at each pixel. Because *r*_*fit*_[*i, j*] is obtained by searching for the optimal τ [*i, j*] from the candidate pool, based on the properties of multiple tests, we introduce the transformation in the first term to ensure that $z_{px}^{\left(fit\right)}\left[i,j\right]$ follows a standard Gaussian distribution.

We then apply the region growing as in Section Active Region Detection on Z-Score Map by Order-Statistics Guided Hypothesis Testing to identify pixels associated with the FIU and assess its statistical significance simultaneously.

#### Refining characteristic curves

We need to refine $X_{m}^{*}$ to remove the imposed constraint of unit L2 norm and to reflect that only pixels in the FIU should contribute to the calculation of the characteristic curve. Hence, denoting the region mask for FIU *m* as *K*_*m*_ we have the final characteristic curve:

(13) $$C_{m}={\sum^{\text{​}}}_{k\in K_{m}}\frac{\beta_{m}^{*}[i_{k},j_{k}]}{\sigma_{0}^{2*}[i_{k},j_{k}]}Y\left[i_{k},j_{k},:+\tau^{*}[i_{k},j_{k}]\right].$$

### Module 3: quality control and quantitative characterization of astrocyte functional status

The accompanying software package provides users opportunity to proof-read and edit the results. A number of informative summary statistics are computed and presented to users for further analysis.

#### Quality control: post-processing and proofreading

In principle, the proposed algorithm has the power to detect any significant Ca^2+^ activity, regardless of what pattern it has. However, not all signals are biologically interesting. Prior knowledge guided post-processing helps users control the quality of results, by expert proofreading or computation-based screening of detected FIUs. The automatic screening filters out a candidate FIU if it does not meet some pre-defined requirements regarding its features obtained from the quantitative analysis illustrated in Quantitative Characterization of Astrocyte Functional Status, such as smoothness of the time-intensity curve, number of peaks or the area of the region.

#### Quantitative characterization of astrocyte functional status

On the basis of the learned model of a time-lapse Ca^2+^ imaging data sample, various quantitative analyses can be conducted to extract features of astrocytic FIUs, which we expect would provide direct and interesting information for biological researches. FASP, by itself, summarizes several basic features as listed below.

##### Ca^2+^ signal curves

The raw time-intensity curves *F*(*t*) = *C*_*m*_(*t*) (Equation 13) are firstly transformed into the description of Ca^2+^ signals: signal-to-baseline ratio of fluorescence (Δ*F*/*F*_0_ = (*F* − *F*_0_) /*F*_0_), where the baseline fluorescence *F*_0_ is estimated as the 10th percentile of the fluorescence levels (intensities) at all the time points during measurement.

##### Amplitudes of Ca^2+^ events

The amplitude of a Ca^2+^ event is calculated as the maximum Δ*F*/*F*_0_ during the transient. Ca^2+^ events are recognized by detecting peaks in the smoothed Δ*F*/*F*_0_ curves.

##### Frequency of Ca^2+^ fluctuations

We define the frequency of Ca^2+^ fluctuations as the inverse of the average duration between two contiguous events. Compared with counting events in a given time period, this definition of frequency ensures more reliable estimation especially in cases where only one or very few events occur during observation, just like what we often see in time-lapse Ca^2+^ imaging data of astrocytes.

##### The half time (T_0.5_)

T_0.5_ of a Ca^2+^ event is calculated using linear interpolation as the time from peak to half amplitude of an event.

##### Area of FIUs

Area of each FIU is represented in both pixels and μ*m*^2^.

##### Average spatial propagation velocity of Ca^2+^ transients in an FIU

Based on the estimated time shift of each pixel's observed curve from the characteristic curve, we can locate all the wavefronts of Ca^2+^ transients in an FIU. Then the intracellular propagation velocity of Ca^2+^ transients is obtained by estimating the average distance between wavefronts.

## Experiments and results

### Real astrocyte calcium imaging data

To validate FASP's effectiveness in analyzing real data, we derived astrocytes *in vitro* from dissociated primary rat culture and human astrocyte induced pluripotent stem cells (hiPSCs). Cells were infected with lentivirus expressing EF1a-GCaMP6, and imaged under a confocal microscope 3 days after infection. Before imaging, the culture medium was removed, and cells were washed with HBSS (Life Tech, with 2 mM Ca^2+^/Mg^2+^) three times. The cells were incubated with HBSS, and imaged using the time series mode (968 ms per image, 2 s interval, for 200 images). Several agonists known to trigger astrocytic Ca^2+^ signaling (Kim et al., 1994; Pasti et al., 1997; Bowser and Khakh, 2004; Fiacco and McCarthy, 2004; Jourdain et al., 2007; Hamilton et al., 2008) were then respectively added to the cells, including ATP, glutamate and metabotropic glutamate receptors (mGluRs). Agonists were added during the first several frames, and incubated for the remaining imaging process.

### Synthetic data

**Figure 4:**
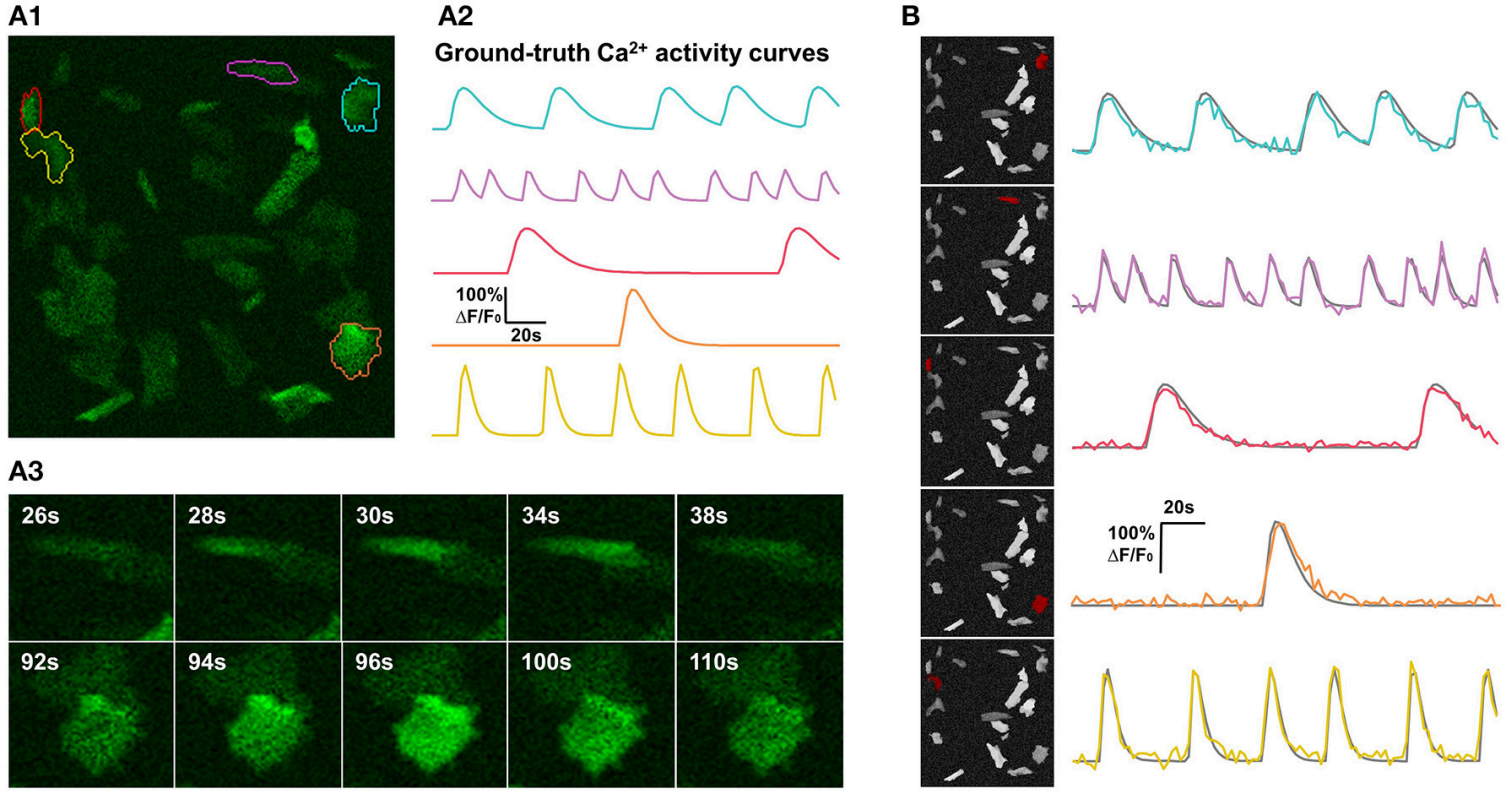
Synthetic data and qualitative test on it. **(A)** One example of synthetic data mimicking time-lapse astrocyte Ca^2+^ imaging data. The images contain only one channel and are represented in green pseudo color. When generating simulated data, critical properties of astrocyte Ca^2+^ time-lapse imaging data are carefully taken into account, including time course pattern, Ca^2+^ propagation pattern, spatial distribution and morphology of FIUs, weak edges of cells, and the gradually fading activities at the boundaries of FIUs. **(A1)** The first frame on which 5 ground-truth FIUs are highlighted using different colors. **(A2)** The ground-truth characteristic curves of the 5 FIUs in **(A1)**, illustrated with corresponding colors. **(A3)** Two image series show examples of Ca^2+^ propagation patterns in the simulated data. **(B)** FASP successfully detected the FIUs. For each detected FIU, the area is highlighted by red color on the neighborhood correlation map for this sample (left), and the corresponding estimated signal pattern is presented using a colored curve, with the ground-truth curve is given in gray for reference (right).

For the purpose of comprehensively and reliably assessing the performance of FASP, we also generated synthetic data sets that simulate Ca^2+^ dynamics in astrocytes but allow for large flexibility of parameter setting (see Figure 4 and Supplementary Video 2 for examples). A synthetic sample was constructed by simulating a spatial distribution of FIUs and Ca^2+^ inactive cells/units, a map of baseline brightness level, characteristic temporal dynamics, intracellular Ca^2+^ propagation patterns of different FIUs, a map of noise level (variance), and a map of non-zero coefficients β_*m*_ in each FIU.

The centroids of FIUs and inactive units were all uniformly distributed in the field of view. We set the proportion of FIUs among the overall units as 1/4 to simulate the spontaneously active cell distribution in *in vitro* Down's syndrome astrocyte iPS cells (Figure 1B). Given the irregular morphology of astrocyte Ca^2+^ FIUs, as well as the complex baseline brightness patterns and weak edges of astrocytes, basic geometric shapes, and artificial baseline distributions do not reflect reality. Thus, we extracted more than four thousand FIU shape and baseline intensity templates from real-world astrocyte imaging data, and, when generating specific simulated datasets, randomly selected from these templates and resized them randomly. Taking diverse potential applications into consideration, we did not impose a prior assumption on the number and density of FIUs in the field of view, but rather tested different combinations in our simulation experiments.

To generate astrocytic FIU characteristic signal curves, we first randomly chose a series of onset times of Ca^2+^ transient events, denoted as $t_{i}^{\left(on\right)},\;i=1,2\ldots n_{evt}$. Then the temporal dynamics were modeled by Mukamel et al. (2009):

(14) $$\begin{array}{c} X_{M}[t]={\sum^{\text{​}}}_{i=1}^{n_{evt}}\left(t-t_{i}^{\left(on\right)}\right)e^{-\left(t-t_{i}^{\left(on\right)}\right)/\eta \;}\cdot I_{t>t_{i}^{\left(on\right)}} \end{array}$$

where $I_{t>t_{i}^{\left(on\right)}}$ is an indicator function with value 1 for the set $\left\{t|\;t>t_{i}^{\left(on\right)}\right\}$; η regulates the duration of transients and varies from FIU to FIU. Then *X*_*M*_[*t*] was rescaled such that the maximum fluorescence signal $\max\left(\frac{\Delta F}{F_{0}}\right)=\left[\max\left(X_{M}\left(t\right)\right)-\min\left(X_{M}\left(t\right)\right)\right]/\min\left(X_{M}\left(t\right)\right)$ is in the range from 0.5 to 4, which is commonly seen in real astrocyte Ca^2+^ imaging data.

Propagation patterns were modeled by setting a distribution on the time delay τ. In each FIU, assuming the Ca^2+^ elevation wave starts from a random pixel and expands to the surrounding pixels at a constant velocity, the time delay of the source pixel was set to be 0 while the other pixels' delays were set proportional to their distances from the source pixel. The map of the noise level was set according to the characteristic curve and the average SNR of each FIU. Following the traditional definition in imaging, signal-to-noise ratio (SNR) is here defined as the ratio of the signal magnitude (maximum intensity change) to the standard deviation of the noise, and then measured in decibels (dB) using the industry standard 20 log rule. The average SNR can either be different from FIU to FIU or kept the same across all FIUs in an imaging sample, depending on different experimental objectives. And the map of non-zero β_*m*_ is designed to reflect the gradually fading strength of activities at the boundaries of FIUs, a critical impact factor for the binarization of z-score maps, which makes the decision of FIU boundaries challenging.

In our simulation experiments, the overall imaging duration was 200 s, with a between-frame time interval of 2 s. Gaussian noises were subsequently added in, in accordance with the given map of noise level.

### Performance metrics

In our experiments, the following metrics were employed to assess the performance of FASP:

#### Recall

Recall is defined as the fraction of ground-truth FIUs that are detected. We say a ground-truth FIU is detected, if there exists one output FIU reported by an algorithm covering more than 50% area of the ground-truth FIU.

#### Precision

Precision is defined as the fraction of reported FIUs that are true FIUs. A reported FIU is thought of as true if it covers more than 50% area of one ground-truth FIU and does not cover more than 10% area of any other ground-truth FIUs. If any ground-truth FIU is falsely divided into two or more parts (reported FIUs), this definition ensures that only one reported FIU will be treated as a correct detection.

#### Fidelity

As a measure of signal estimation quality, the fidelity of a learned characteristic curve is defined as the Pearson's correlation coefficient between the learned curve and the ground-truth characteristic curve.

#### Area accuracy

Any reported FIU considered as true uniquely indicates one ground-truth FIU. The detection accuracy of the reported FIU is further evaluated by area accuracy, defined as the fraction of the corresponding ground-truth FIU that is covered by this reported FIU.

### FASP can handle datasets of heterogeneous and low SNR values

**Figure 5:**
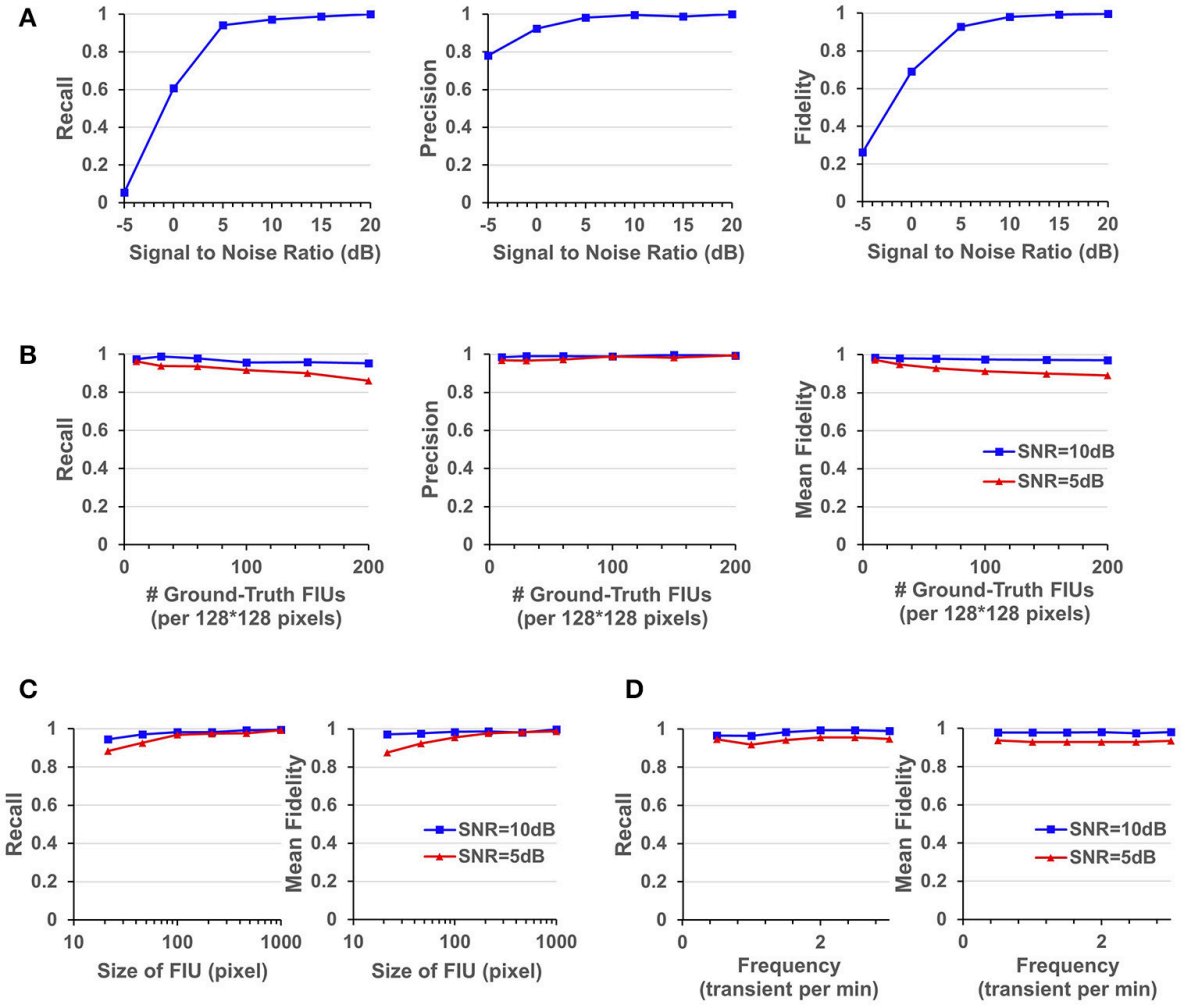
Quantitative performance evaluation under different parameter settings. **(A)** FASP's performance under situations with different SNR values. **(B)** The influence of density of FIUs. **(C)** Evaluate the robustness regarding spatial sizes of FIUs. **(D)** Robustness regarding temporal pattern of characteristic curves, reflected by the temporal frequency of transients.

The performance of FASP was evaluated as a function of SNR using simulated data (Figure 5A). For each SNR value, 25 videos were generated with 40 FIUs per video. Ca^2+^ dynamics were randomly simulated using Equation (14), and the propagation speed was also uniformly sampled from the interval [1, 30] pixels per frame. Since FIUs were independently created, we evaluated detection performance averaging over all FIUs which were considered as individual detection events.

As one would expect, performance improves with increasing SNR. But it is notable that the algorithm works quite well even when the SNR is 5 dB, getting an overall recall (in terms of all FIUs) of 0.941, an overall precision of 0.982, and a mean fidelity of 0.928 with more than 90% of learned characteristic curves having fidelities >0.9. When the SNR is higher than 5 dB, all the three performance scores tend asymptotically to 1. A side note is that on the real data, the typical SNR is between 3 and 10 dB.

### FASP is robust to various FIU spatial patterns and temporal activity patterns

Astrocyte Ca^2+^ signaling has very complex spatial and temporal patterns. Firstly, the morphological heterogeneity of astrocytes are commonly observed in studies (Oberheim et al., 2012). And since an FIU is usually only a part of an astrocyte, astrocytic FIUs could be even more irregular in terms of shape or size (Figure 1 and Supplementary Video 1). Secondly, though some typical astrocytic Ca^2+^ transient models have been built, the characteristic curves still heavily differ from each other in overall pattern, especially in different experiments.

FASP makes no assumption about FIUs' morphology, propagation speed, or any parametric model for the characteristic time courses. The morphology of FIUs and curves' patterns are learned from the data. These properties endow FASP with excellent flexibility and stability with these complex impact factors. As the synthetic data were generated using shape templates from real data, good overall performance shown in Section FASP Can Handle Datasets of Heterogeneous and Low SNR Values already demonstrates that FASP can work with various morphology patterns of FIUs. In addition, with image size, SNR, and number of FIUs fixed, and with all other impact factors randomly sampled from given distributions, we investigated FASP's performance as response to size of FIU and frequency of Ca^2+^ transients. Figure 5C suggest the impact of FIU size is not substantial as long as an FIU covers more than ~ 10 pixels. And Figure 5D shows that the frequency has almost no impact on the algorithm's performance.

### FASP is robust to various FIU density and total number in the field of view

Due to different experimental conditions and different requirements from applications, fluorescence imaging data of astrocyte Ca^2+^ signaling could exhibit considerable variation in FIU population density and the total number of FIUs in the field of view. Generally speaking, the more FIUs one has, the more difficult the analysis is. However, it is a fundamental requirement for automatic analysis of large-volume datasets to simultaneously and accurately detect hundreds or even more FIUs without prior knowledge of the number of FIUs. Quantitative assessment on simulation datasets shows that FASP has flexibility with number and density of FIUs in the field of view and can thus successfully meet this demand.

In simulations with fixed image size, fixed SNR, random FIU size (≥10 pixels) and random propagation speed, samples consisting of different numbers of FIUs were tested. The impact factor of interest here is the population density of FIUs. As shown in Figure 5B, our algorithm's recall mildly drops as the population density of FIUs increases, but still retains good performance (mean fidelity of 0.891, recall of 0.861, and precision of 0.994) even with more than 200 FIUs in a 128^*^128-pixel video and SNR of 5 dB. Most errors were caused by adjacent FIUs whose ground truth characteristic signals are intrinsically similar to each other and hence difficult for algorithms or even humans to distinguish. The denser the FIUs are in the field of view, the more likely FIUs connect spatially to each other, and hence the more difficult it is to distinguish them.

**Figure 6:**
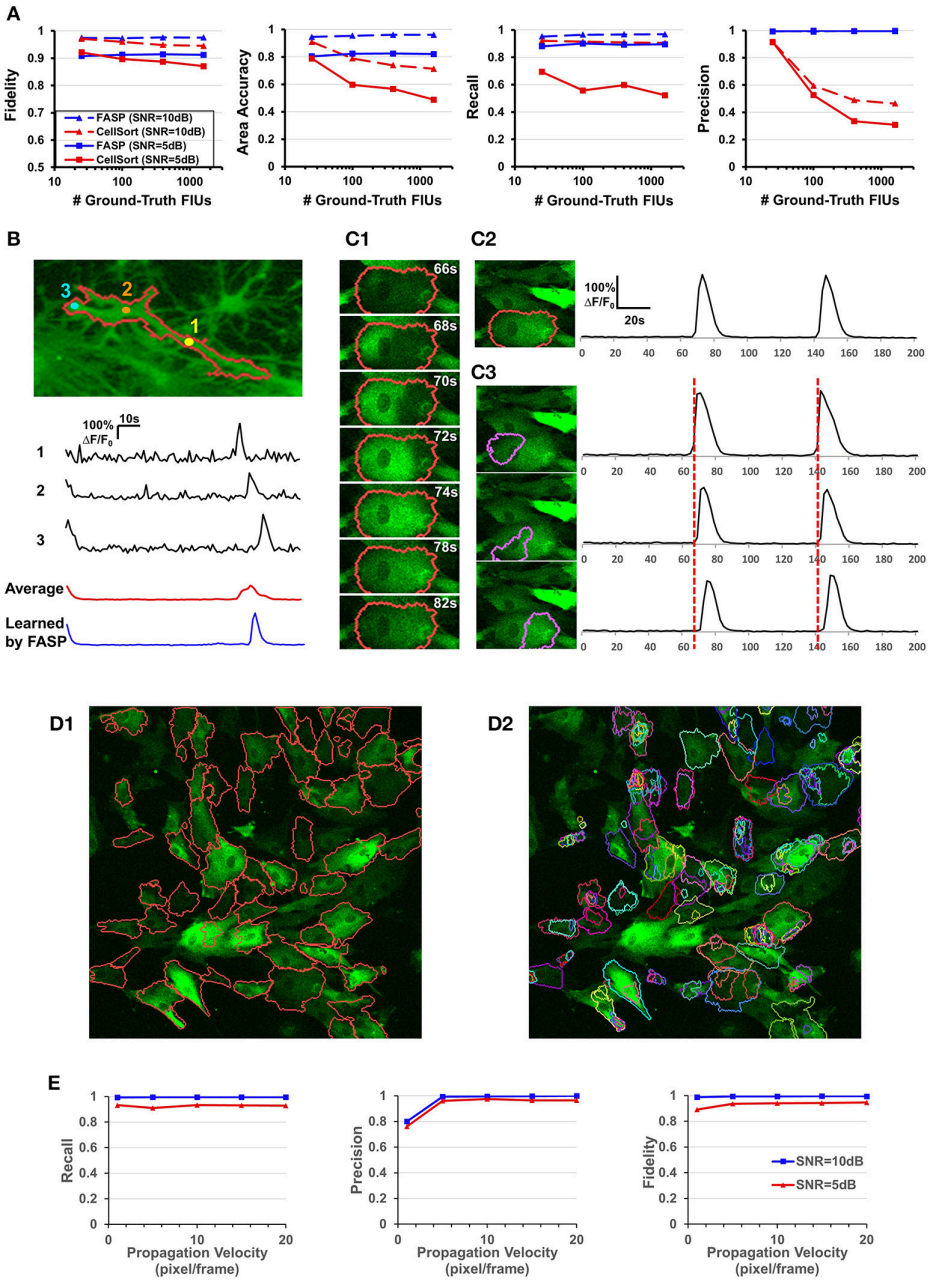
Well modeling the propagation of intracellular Ca^2+^ transients endows FASP with good performance. **(A)** Quantitative performance comparison between FASP and CellSort (Mukamel et al., 2009), a popular algorithm that works well on data with no propagation phenomena. FASP outperforms CellSort significantly, suggesting the importance of modeling propagation phenomena in the analysis of astrocytes. **(B)** FASP resolves the negative impact of propagation phenomena on the estimation of Ca^2+^ dynamic curves. The first three curves are the observed time courses at pixels in the same FIU, showing time lags due to propagation. The red curve is the average of observed curves at all pixels in this region without considering the time lags, and hence the curve is distorted from the observed ones, with much longer rise time and decay time of Ca^2+^ transients. The blue curve is the fitted signal curve given by FASP. **(C1)** Selected frames from a time-lapse data sample show apparent propagation of Ca^2+^ elevation. **(C2)** The results produced by FASP, including the spatial map of one FIU and the corresponding time curve. **(C3)** The sorting results produced by CellSort, related to the FIU highlighted in **(C2)**. The connected domain was falsely split and identified as three differently functioning ROIs. Their fitted curves are actually just the same one, if time lags are taken into account. **(D)** Comparison between FASP and CellSort on a real data sample of astrocytes derived from human induced pluripotent stem cells (hiPSCs). **(D1)** FIUs identified by FASP highlighted by carmine boundary lines on the first frame of the original image stack. To better check whether the detection is correct, please see Supplementary Video 1. **(D2)** Regions of Interest (ROIs) identified by CellSort highlighted by boundary lines on the first frame. Different ROIs were labeled using different colors to clearly show the overlap between ROIs. Please refer to Supplementary Video 3 to check the performance of CellSort. Many FIUs were falsely separated into parts by CellSort, due to the violation of within-ROI synchronization of Ca^2+^ elevations. Some FIUs were detected multiple times, as a consequence of blind decomposition of signal matrix by CellSort. **(E)** The impact of propagation velocity on FASP's performance.

The second set of simulation experiments targeted the influence of the total number of FIUs given the FIU population density. We did simulations with fixed FIU intensity in the field of view, fixed SNR, random FIU size (≥10 pixels) and random propagation speed. Samples of different image sizes were generated such that they contained 25, 100, 400, and 1,600 FIUs. The blue lines in Figure 6A show the very promising performance of FASP on data with a large number of FIUs. No matter the SNR is 5 or 10 dB, good performance is preserved as the total amount of FIUs dramatically increases.

### Well modeling the propagation of Ca^2+^ transients endows FASP with good performance

One of the key challenges in astrocyte Ca^2+^ activity analysis is the pervasive phenomena of slow signal propagations of intracellular Ca^2+^ transients. Figures 1B,C give two real-world examples of this phenomenon. Figure 6B shows time lags between pixels in a real astrocyte functional unit, as a result of the slow propagation. All these examples demonstrate the special nature of astrocyte Ca^2+^ time-lapse imaging data, which, we anticipate, rules out algorithms without specific design for the complex phenomena. On the contrary, by explicitly modeling the propagation, we expect that FASP gains the power to effectively eliminate the ill effects of it and to correctly capture the actual astrocyte Ca^2+^ dynamic signals.

To the best of our knowledge, designed for astrocyte or any other cell types, no existing method of time-lapse Ca^2+^ image analysis has so far made any effort to tackle the problems caused by the propagation phenomena. To validate the necessity of explicitly modeling the propagation phenomena, we compared FASP with “CellSort” (Mukamel et al., 2009), one of the most popular Ca^2+^ dynamic detection algorithms that work quite well on neuronal Ca^2+^ imaging data. Ignoring the effect of propagation, CellSort makes a basic assumption of within-FIU synchronization, which approximately holds true for neuronal Ca^2+^ data. The violation of this basic assumption in astrocyte time-lapse Ca^2+^ image data constitutes fundamental problems for CellSort in detecting astrocytic FIUs. On the one hand, as the possible time lags between observed signals in different parts of an FIU are neglected, only pixels with almost the same phase of signal are going to be clustered together. Put differently, pixels in one ground-truth FIU (Figure 6C1) could be falsely detected as several FIUs, as shown in Figure 6C3. In contrast, FASP successfully detected the FIU as a whole (Figure 6C2). On the other hand, under the assumption of within-FIU synchronization, the learned characteristic curve is essentially a weighted average over all pixels' signal curves without considering the time lags between pixels, leading to distortion of the characteristic curve (Figure 6B), which makes the subsequent analysis misleading. The characteristic curve estimated by FASP, however, well matches the ground truth (Figure 6B). For comparison between FASP and CellSort regarding average performance, we tested them on both real data and synthetic data. Figure 6D and Supplementary Videos 1, 3 together give a comparison on real data, supporting our conclusion that CellSort would often falsely split one FIU into several, due to the impact of pervasive propagation phenomena. Because of the lack of ground-truth for real data, we decide one FIU should not be separated into parts if we see the same temporal Ca^2+^ fluctuation pattern in all parts of this FIU, without propagation or only with spatially continuous and smooth propagation (see Supplementary Videos 1, 3). Differently from CellSort, FASP correctly detected the FIUs. Quantitative comparison was done by testing CellSort on the same synthetic dataset generated in Section FASP is Robust to Various FIU Density and Total Number in the Field of View, with a common and fixed FIU density in the field of view but different image sizes. Figure 6A clearly shows that our algorithm is superior to CellSort. CellSort reported a lot of redundant units because it allows for totally or partially overlapping FIUs and it falsely separates real FIUs into sub-regions. The false separations also give rise to a smaller area accuracy. Besides, the fidelity of curves estimated by CellSort is lower. In cases with SNR of 5 dB, CellSort missed a lot of FIUs, while our method still found around 88% ground-truth FIUs, suggesting a much better robustness to large noises. All these results emphasize the importance of well-modeling the propagation phenomena in astrocyte Ca^2+^ image analysis.

Then we did quantitative analysis to assess the robustness of FASP to the severity of propagation phenomena. FASP's performance was evaluated as a function of Ca^2+^ propagation velocity. With other parameters randomly sampled from a given distribution, we generated synthetic data using different propagation velocities. The performance is shown in Figure 6E. When the intracellular calcium transients propagate at a velocity of 1 pixel per frame or faster, the velocity generally has little impact on the performance. A natural corollary of this observation is that our algorithm is compatible with fast-propagation applications such as neuronal Ca^2+^ dynamic detection. When the propagation velocity is slower than 1 pixel per frame, the precision drops a little, perhaps because the differences between adjacent pixels are larger and hence the pixel-wise neighborhood correlations are smaller, although we did not observe such slow propagation in real data.

### Case study: analyzing agonist-induced Ca^2+^ signaling in rat astrocytes

**Figure 7:**
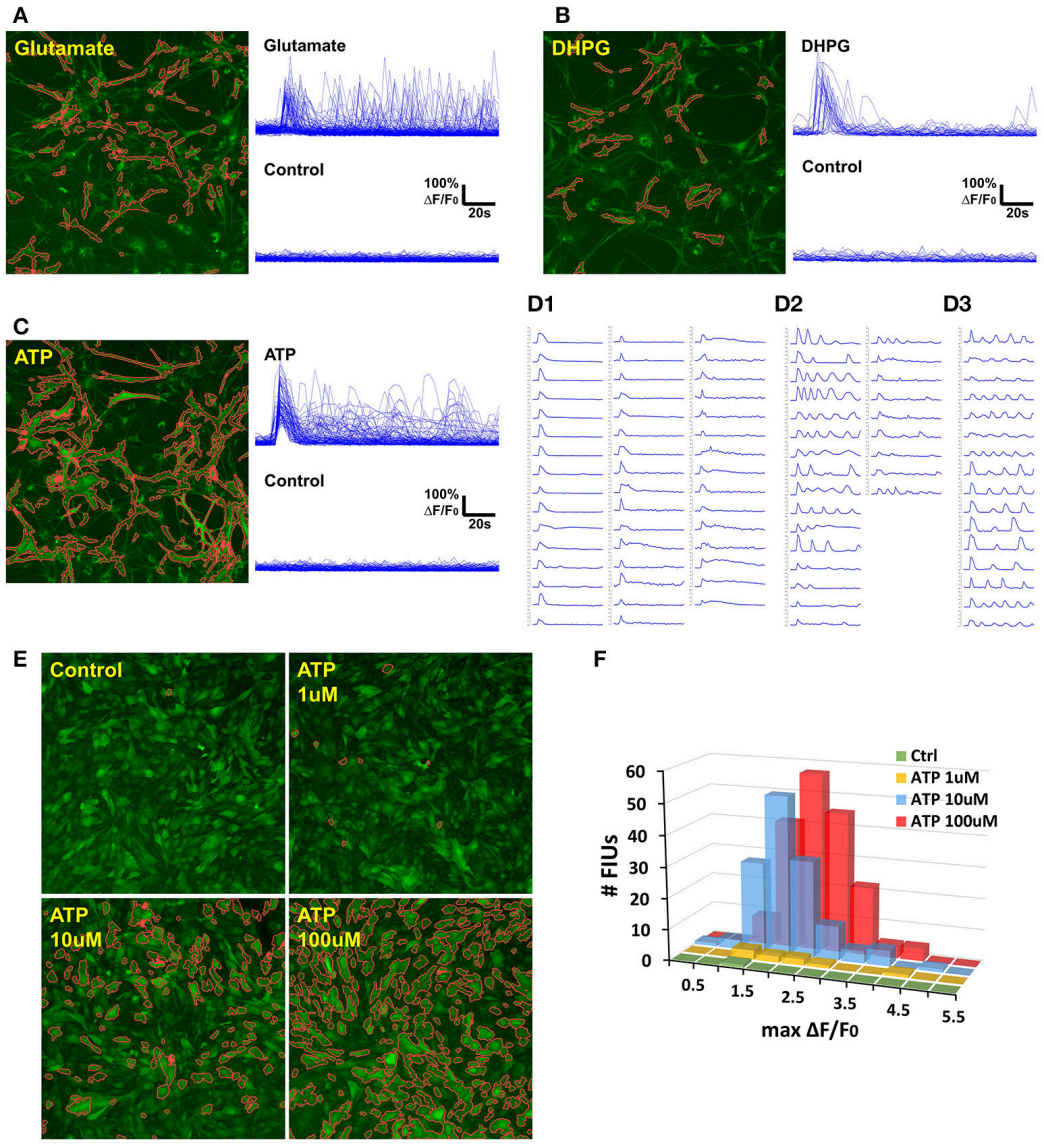
Case study: analyzing agonist-induced Ca^2+^ signaling in *in vitro* rat astrocytes. **(A)** 129 FIUs with glutamate-evoked Ca^2+^ signals, identified by FASP. Left: the FIUs' spatial positions were highlighted with carmine edges. Right: the learned characteristic signal curves of the FIUs shown on the left. Each curve in the control group is the average time course over pixels in the corresponding glutamate-induced FIU detected in the case sample. **(B)** 33 FIUs with DHPG-evoked Ca^2+^ signals identified by FASP. **(C)** 88 FIUs with ATP-evoked Ca^2+^ signals identified by FASP. **(D)** The ATP-evoked Ca^2+^ signals in **(C)** listed individually in **(D)**, from which different patterns can be recognized: **(D1)** FIUs that responded only once after the agonist was added, **(D2)** FIUs with evoked oscillations of decreasing frequency or decreasing amplitude, and **(D3)** FIUs with evoked oscillations of generally constant frequency and constant amplitude. **(E)** FASP captured the correlation between ATP dose and the number of responding FIUs. The FIUs detected by FASP are highlighted using carmine contours on each sample. **(F)** The relationship between ATP dose and the histogram of Ca^2+^ activity amplitudes (maximum Δ*F*/*F*_0_).

We applied FASP to analyse the calcium dynamics of rat hippocampal astrocytes in response to agonist treatment (Figure 7). When ground truth is absent for validation, data of stimuli-triggered activities present an alternative way to test data analysis methods. Astrocytic Ca^2+^ signaling can be triggered by the excitatory neurotransmitter including ATP and glutamate through P2Y1 receptors and metabotropic glutamate receptors (mGluRs) respectively (Kim et al., 1994; Pasti et al., 1997; Bowser and Khakh, 2004; Fiacco and McCarthy, 2004; Jourdain et al., 2007; Hamilton et al., 2008). 3,5-dihydroxyphenylglycine (3,5-DHPG), a potent agonist of group I mGluRs (Wisniewski and Car, 2002), including mGluR1 and mGluR5, can evoke astrocyte Ca^2+^ elevations in the astrocyte soma and fine processes (Zur Nieden and Deitmer, 2006). We thus monitored the calcium dynamics in response to ATP, glutamate, and DHPG on cultured rat astrocytes, respectively, followed by FASP analysis to detect the evoked Ca^2+^ activities. For each sample, the astrocytes were imaged twice, before and after the agonist was added in.

To assess the accuracy of automatic analysis, we first manually labeled FIUs in all the control/case samples, and compared them with the outputs of FASP. It turned out that the FIUs automatically detected by FASP were very consistent to those manually labeled, but had more complex and accurate contours. FASP was able to successfully detect some FIUs that have low basal fluorescence and therefore were neglected by manual labeling. We confirmed those neglected FIUs by double-checking those areas by purposefully applying transformations which facilitate human vision. The detected FIUs included whole somas, long processes, and smaller sub-regions within cells (probably microdomains; Figures 7A–C,E). Connected FIUs were successfully detected as separate units.

The control samples for all the three agonists showed almost no spontaneous activity before the application of the agonists, but a large number of FIUs were activated with the agonists applied separately (Figures 7A–C). One hundred and twenty-nine induced FIUs were detected after glutamate was applied, covering 28.45% of the area of all astrocytes (active/silent) in the sample, while ATP alone induced 88 FIUs that covered 57.27% of the area. These observations suggest strong effects of these agonists. DHPG showed a relatively milder effect, activating 33 FIUs, 18.50% of the area of overall astrocytes. Agonist-evoked Ca^2+^ was well-captured by the algorithm. The agonists were added in during the first several frames of imaging, and an obvious burst of Ca^2+^ elevations can be seen in all the three samples with agonists. Accordingly, characteristic curves reported by FASP show a common Ca^2+^ increase shared by the majority of FIUs at about the 6th frame (Figures 7A–C). The estimated half-rise time ranges 1–6 s and half-decay time ranges 4–10 s, which is consistent with what was previously reported about Ca^2+^ dynamics in response to ATP stimuli (De Pitta et al., 2012). The response to ATP is asynchronous. From the curves of induced Ca^2+^ signals (Figure 7D), we can recognize FIUs that responded only once right after the agonist was added, FIUs with evoked oscillations of generally constant frequency and constant amplitude, and FIUs with evoked oscillations of decreasing frequency or decreasing amplitude. Similar patterns were also observed in other studies, suggesting that ATP is a modulator for both frequency and amplitude (Cornell-Bell et al., 1990).

We next test the accuracy of FASP's outputs in detecting dose response to agonists. When ATP of different doses is added, the number of FIUs responding to the agonist stimuli is supposed to increase as the dose increases. Figure 7E and Supplementary Videos 4–7 gives a comparison among the detected responses to no ATP (control), 1, 10, and 100 uM ATP. The control sample only contained one FIU with spontaneous activity; 1 uM ATP induced 9 FIUs; 10 uM stimulated 136 FIUs; while the field of view was full of responding FIUs (182 FIUs) after 100 uM ATP was applied. The peak response to different doses is also reflected in the histogram of estimated Ca^2+^ activity amplitude (maximum Δ*F*/*F*_0_; Figure 7F). The higher the dose is, the higher the histogram is (because more FIUs responded), and the more large-amplitude activities are observed. These results suggest that FASP successfully captured the association between the strength of stimulated activities and agonist dose.

### The binarization method based on order-statistics theory is effective

**Figure 8:**
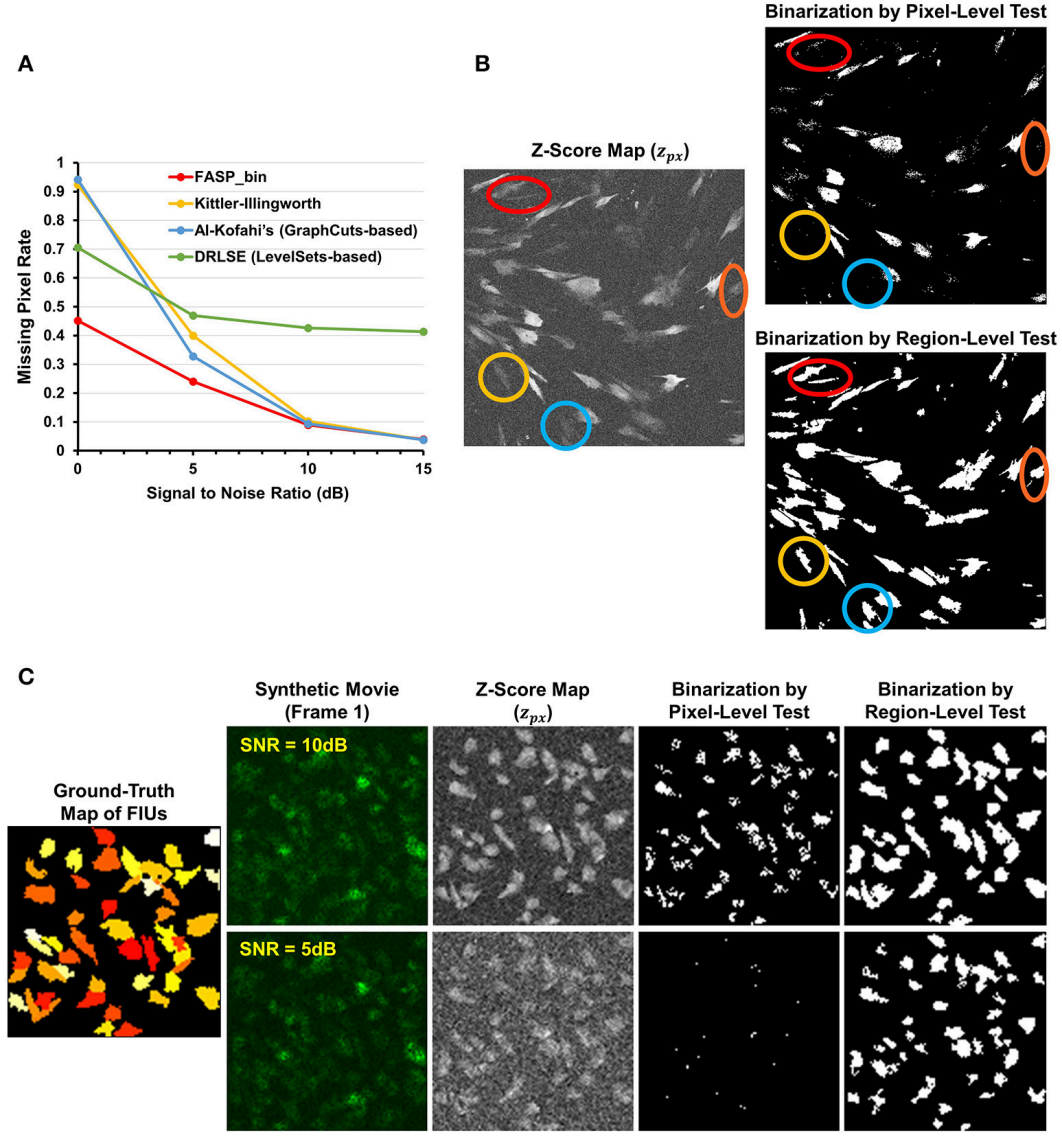
Effectiveness of z-score map binarization method based on order-statistics guided hypothesis testing. **(A)** Comparison of missing pixel rate, as a function of SNR, among peer binarization algorithms. Except for SNR, all parameters were randomly sampled to generate the simulated data. Our method has strong power to binarize z-score maps and outperforms others especially in low-SNR cases. **(B)** A real data sample of *in vitro* astrocytes shows that accumulated information in a region helps to enhance the statistical power of tests and, consequently, the performance of identifying FIUs. Two hypothesis testing based binarization methods were compared: (1) a right-tailed test on each pixel using the pixel-level z-score; (2) a right-tailed test on any possible connected domain using the region-level z-score (accelerated by our heuristic region search process without loss of information). Colored circles highlight a portion of weak regions which are completely neglected by the pixel-level tests but well-identified by the region-level method. **(C)** An example of the same comparing experiments on synthetic data. Data samples were generated with SNR being 5 or10 dB, respectively, and the two binarization methods were compared in both cases. The region-level tests gave much better detection performance. Panels **(B,C)** provide one explanation for the good performance of our z-score map binarizatoin method.

The z-score maps generated from astrocyte Ca^2+^ time-lapse images usually possess the following characteristics which make the binarization task difficult: (1) large noises and small foreground/background contrast due to low-SNR in input image stack data; (2) weak and blurred edges of foreground areas; and (3) uneven intensity distributions across different foreground regions. Taking advantage of the statistical nature of z-score maps, we based our method for binarizing z-score maps on order-statistics guided hypothesis testing (Section Active Region Detection on Z-Score Map by Order-Statistics Guided Hypothesis Testing). It was demonstrated by experiments that our method has strong power to binarize z-score maps and outperforms many other binarization techniques especially in low-SNR cases (Table 1 and Figure 8A).

**Table 1:** Comparison of binarization methods on the z-score map.

	**Misclassification rate**	**Recall**	**Precision**	**F-measure**
FASP-bin	**0.0291** ± **0.0096**	**0.8257** ± **0.0487**	0.9847 ± 0.0061	**0.8976** ± **0.0306**
Kittler-Illingworth thresholding	0.0551 ± 0.0163	0.6480 ± 0.1053	0.9879 ± 0.0052	0.7779 ± 0.0821
Al-Kofahi's (Graph Cuts based)	0.0517 ± 0.0146	0.6652 ± 0.0943	**0.9963** ± **0.0028**	0.7940 ± 0.0705
DRLSE (Level Sets based)	0.1233 ± 0.0579	0.5390 ± 0.1272	0.7212 ± 0.2255	0.5853 ± 0.1013

*Each pixel is classified/labeled as either a foreground pixel or background pixel, and the four metrics are based on the proportion of correctly classified pixels or misclassified pixels in an image. The evaluation scores in the table are presented in the format “average ± SD,” where SD is the standard deviation calculated over multiple runs of simulation. Bold fonts indicate the best result in terms of each performance measure. FASP-bin is the method developed in this study. The synthetic data here were generated with all parameters randomly sampled from given distributions that reflect typical parameter values in real data. All methods were tested on the same datasets*.

Literature shows several methods that are most often applied to binarize grayscale microscopy images of cells (Meijering, 2012): thresholding, watershed transformation, active contour methods, and graph cuts based algorithms. Unfortunately, due to the special characteristics and difficulties of our data, some key assumptions explicitly or implicitly required by these methods are not satisfied, such as assumptions of separable foreground/background intensity distributions, intensity homogeneity within foreground/background regions, and sharp intensity change at the boundaries between foreground and background. Experimental results on synthetic data confirmed the concern that the violation of these strong assumptions makes existing methods intrinsically problematic to solve our problem. Watershed approach typically relies on predefined markers of foreground and background areas which are unavailable in our problem. For the other three categories of techniques, we selected one representative automatic algorithm from each of them and quantitatively evaluate their performance: Kittler-Illingworth thresholding (Kittler and Illingworth, 1986), the best among 40 thresholding methods according to a survey given by Sezgin (Sezgin, 2004); Distance Regularized Level Set Evolution (DRLSE) (Li et al., 2010), which has been applied to cell segmentation problems (Dzyubachyk et al., 2010); and Al-Kofahi's method (Al-Kofahi et al., 2010), a well-accepted graph cuts based algorithm with fully automatic initialization. Parameters were tuned by experiments, with the best performance achievable reported here. According to the experimental results (Table 1 and Figure 8A), our hypothesis testing based region growing method (“FASP-bin”) has the best overall binarization performance among the four, in terms of both misclassification rate and F-measure. Though the precision of our method is slightly lower than that of Kittler-Illingworth thresholding and Al-Kofahi's, the difference is quite small (<0.015) and their recall scores are much lower than ours (>0.160). Figure 8A further reveals that our method shows strength and superiority mainly in low-SNR cases. The missing pixel rates of the other three methods increase tremendously as SNR decreases from 5 to 0 dB.

One key design in our method that makes a critical contribution to this promising performance is the utilization of neighborhood pixels' relationships in hypothesis testing. Specifically, instead of testing each pixel in isolation, we designed a region-level z-score (Equation 4) and tested one candidate region as a whole. In this way the statistical power of test is much enhanced, since information from connected pixels can confirm each other and thus jointly provide us with higher confidence in decisions. On both real data (Figure 8B) and synthetic data (Figure 8C), we compared two hypothesis testing based methods for binarization: (1) a right-tailed test on each pixel using the pixel-level z-score; (2) a right-tailed test on any possible connected domain using the region-wide z-score (accelerated by our heuristic region search process without loss of information). In both methods, pixels (groups of pixels) of interest were claimed positives using a significance level of 0.05. Commonly seen in bioimaging data, the z-score is not homogeneous even within a single FIU. Pixel-level tests discard low scoring pixels, resulting in a coarse and incomplete map, while region-level tests retain these pixels because of their neighbors. More importantly, because of low expression level of fluorescence protein, it is common that some FIUs' signals are too weak that in-FIU pixels can hardly be distinguished from random noise pixels in terms of intensity. But weak signals in a group of connected pixels together reveal the non-randomness, which allows region-wide tests to detect them.

### Neighborhood correlation in residual signals enables a sequential FIU identification

In the sequential process of identifying FIUs in an active region, as discussed in Section Module 2: Identifying FIUs and Extracting Characteristic Curves, at each step only one FIU *m* (with characteristic curve *X*_*m*_) is modeled and all the remaining FIUs are “hidden.” We designed a score $z_{px}^{\left(fit\right)}$ (Equation 12), aiming to distinguish pixels in FIU *m* from pixels in other FIUs. One important innovation in our framework is the realization that correlation between neighboring pixels' residual signals can be used as a measure of signals that cannot be explained by *X*_*m*_ and thus an indicator of the existence of other FIUs. Neglecting this information may lead to inaccurate boundaries between FIUs, or even to total failure in separating different FIUs.

**Figure 9:**
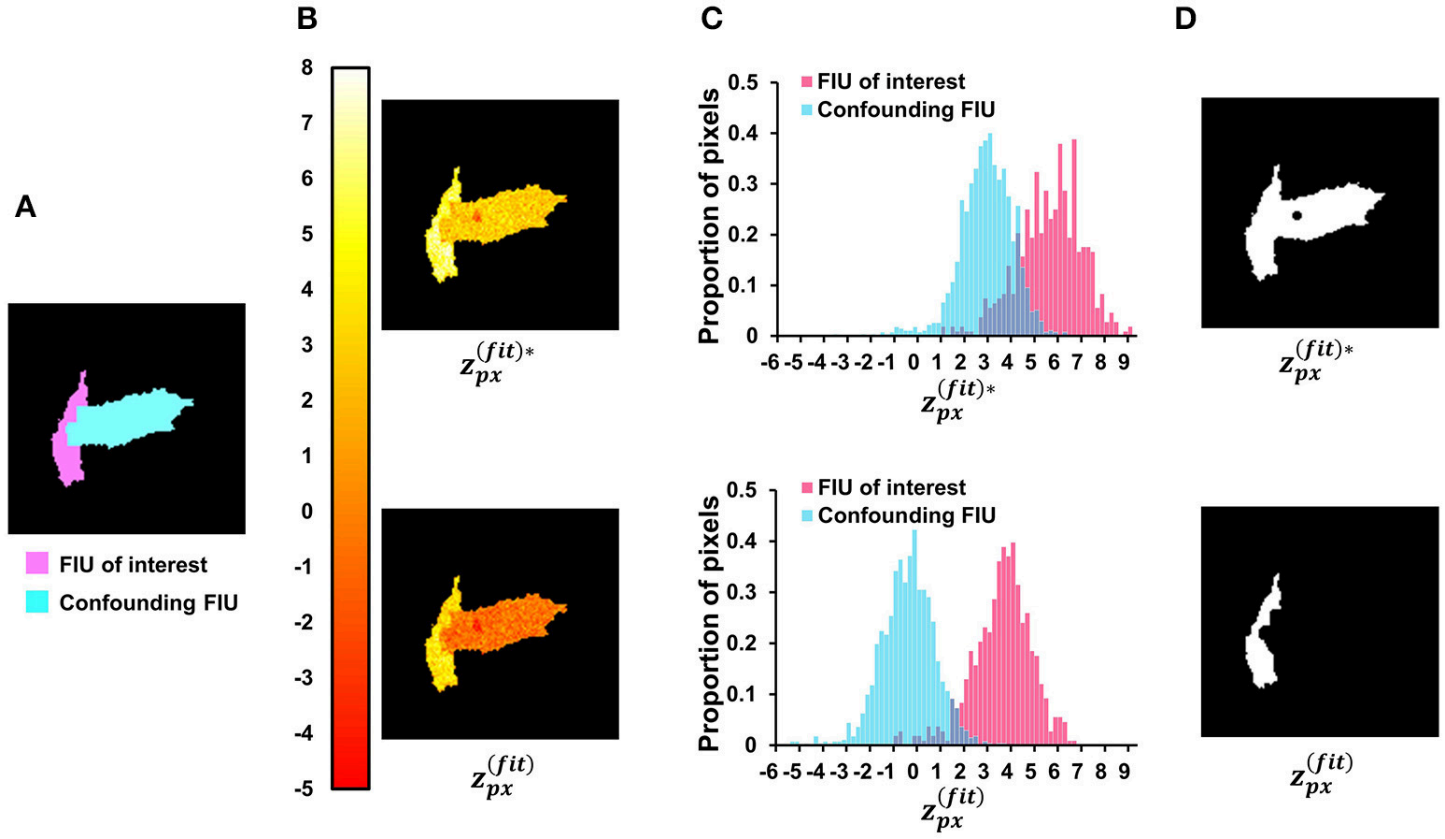
Investigate the role of neighborhood correlation of residual signals in score $z_{px}^{\left(fit\right)}$. **(A)** The ground-truth FIU map of a synthetic data sample. **(B)** Given SNR = 5 dB and correlation coefficient between two FIUs' characteristic curves as 0.4, the color map shows the spatial distribution of $z_{px}^{\left(fit\right)}$ (Equation 12—with penalty term for neighborhood correlation in residuals) and $z_{px}^{\left(fit\right)*}$ (Equation 15—without the penalty term). **(C)** The normalized histograms of $z_{px}^{\left(fit\right)}$ and $z_{px}^{\left(fit\right)*}$, where $z_{px}^{\left(fit\right)}$ shows a better contrast between the FIU of interest and the confounding FIU. **(D)** The results of binarization on $z_{px}^{\left(fit\right)}$ and $z_{px}^{\left(fit\right)*}$ maps. In the up-subfigure of **(D)**, $z_{px}^{\left(fit\right)*}$, ignoring the neighborhood correlation in residual signals, leads to false merging of two distinct FIUs.

We verify the important role that neighborhood correlation of residual signals plays by inspecting the difference between Equation (12) and the following fitness definition:

(15) $$z_{px}^{(fit)*}[i,j]=\Phi^{-1}(\Phi^{2U_{\tau }}(F(r_{fit}[i,j]))),$$

which is linearly related to the first term of $z_{px}^{\left(fit\right)}$ and does not include the information about residual signals. Figure 9 provides an indicative example comparing the two scores. Given two simulated FIUs whose characteristic curves are different but correlated (correlation coefficient = 0.4), the ground truth characteristic curve of one FIU is used to calculate the scores at all pixels. The results support our expectation that $z_{px}^{\left(fit\right)}$ yields better contrast than $z_{px}^{\left(fit\right)*}$. After applying our z-score map binarization method to both maps, the FIU of interest was correctly identified from $z_{px}^{\left(fit\right)}$ map but falsely merged with the confounding FIU using $z_{px}^{\left(fit\right)*}$ map. Generally, the lower the correlation between the two curves, the better the contrast. But in all cases, $z_{px}^{\left(fit\right)}$ performs better. The specific design of $z_{px}^{\left(fit\right)}$ (Equation 12) is one of the keys to the good performance of FASP.

### Alternative design of neighborhood correlation score

**Figure 10:**
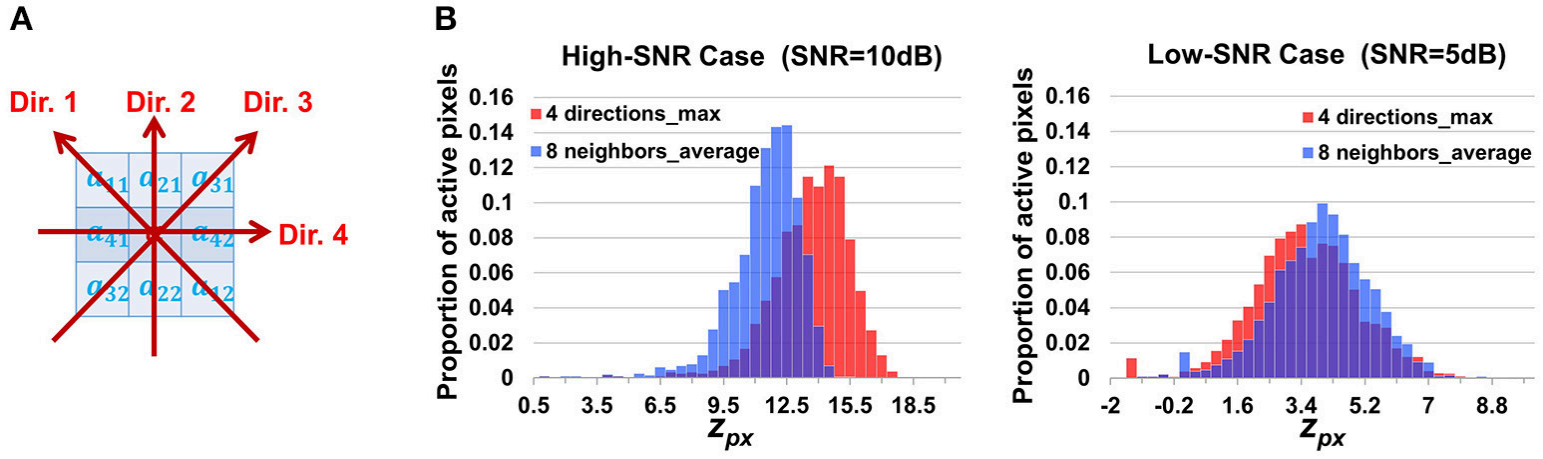
Discussion on the design of neighborhood correlation z-score. **(A)** The four directions used to calculate the alternative neighborhood correlation at a pixel (Equation 16). **(B)** The comparison between two definitions of neighborhood correlation score, showing the histograms of z-scores at active pixels. Since our design guarantees that both scores follows standard Gaussian distribution at silent pixels, the higher a z-score is at active pixels, the better the contrast between active/silent (foreground/background) pixels, and the better the score definition is. “8 neighbors_average” in the legend indicates definition given by Equations 2, 3; while “4 directions_max” corresponds to Equation (16), (17). These results demonstrate that when the SNR is large, “max” is better, while when the SNR is low, “averaging” is better.

When calculating the neighborhood correlation score using Equation (2), we focused more on controlling the damaging effects of noises in low-SNR cases and ignored the potential time lags between adjacent pixels. To examine the impact of this ignorance and investigate FASP's applicability to various situations, we have also considered an alternative neighborhood correlation score, taking propagation phenomena into account. Based on the fact that the signals are always synchronized along the surface orthogonal to the propagation direction, we calculate the neighborhood correlation at pixel [*i, j*] as

(16) $$\begin{array}{l} \begin{array}{c} \;r_{sc}[i,j]=\max_{d=1,2,3,4}cor \end{array}\\ \;\begin{array}{c} \left(Y[i,j,:],\frac{Y[i_{d_{1}},j_{d_{1}},:]+Y[i_{d_{2}},j_{d_{2}},:]}{2}\right), \end{array} \end{array}$$

where *cor*(,) is the Pearson correlation coefficient, *d*_1_ and *d*_2_ are the first and second pixels along the direction *d*. For each pixel, there are four directions to search as shown in Figure 10A. Though we do not know the propagation direction a priori, the maximum value among the four approximately represents the correlation between the pixel and its synchronized neighbors. Accordingly we adjust the definition of the z score of the neighborhood correlation. Considering that the correlation map was obtained by taking the maximum value among four directions, we make a transformation

(17) $$\begin{array}{c} z_{px}[i,j]=\Phi^{-1}(\Phi^{4}(F(r_{sc}[i,j]))), \end{array}$$

where Φ(·) denotes the standard Gaussian CDF and *F*(·) is the normalized Fisher transformation defined in Equation (3). Based on the properties of the Fisher transformation and of extreme order statistics, it can be shown that *z*_*px*_[*i, j*] defined in Equation (17) also follows a standard Gaussian under the null hypothesis that the pixel [*i, j*] has zero β_*m*_ for all *m*. This design is expected to be better to cope with propagation but less powerful to suppress noises, as a result of not taking full use of the eight neighboring pixels but only utilizing two of them in calculating the correlation.

Simulation experiments were conducted to compare Equations (2) and (16) under different ground-truth SNR settings (Figure 10B). Propagation velocities were randomly sampled from a wide range, mimicking real data. The results show that, when SNR is high, Equation (16) leads to better contrast between active and silent pixels than Equations (2) and (3) (*p* < 2.2 × 10^−16^ by Wilcoxon signed rank test). This suggests that the special design in Equation (16) successfully works to suppress the effect of Ca^2+^ propagation. On the other hand, when the SNR is low, the opposite is observed: Equations (2) and (3) assign larger *z*_*px*_ to active pixels than Equations (16) and (17) do (*p* < 2.2 × 10^−16^ by Wilcoxon signed rank test). This supports the notion that, in low-SNR cases, though Equation (16) still models the propagation better, the benefit of restraining noises dominates. Since astrocyte Ca^2+^ imaging data typically has low SNR, we decided to adopt Equation (2). In applications where SNR is basically high while the propagations are extremely slow, Equation (16) can be applied instead.

## Discussion

### Necessity of specific tools for analyzing time-lapse astrocyte Ca^2+^ imaging data

To the best of our knowledge, no tool has been developed for fully automatic analysis of astrocytic signaling from time-lapse Ca^2+^ images. However, many analytical tools have been proposed to analyse similar imaging data of other types of cells, especially neurons. Is it necessary to design new analytical tools specifically for astrocytes? In addition to the discussion about CellSort in Section Well Modeling the Propagation of Ca^2+^ Transients Endows FASP with Good Performance, our survey also shows that neuron-targeted algorithms can hardly be directly generalized to work on astrocyte Ca^2+^ imaging data, demonstrating the needs for new designs.

The earliest and most elementary types of neuron-targeted methods are manual (Dombeck et al., 2007; Göbel et al., 2007) and semi-automated approaches (Junek et al., 2009; Peters et al., 2014) that cannot be applied to large-scale data sets and which are also biased by subjective judgments. Another group of early stage methods is based on detecting a region of interest (ROI) on temporally averaged intensity images without considering the time course information (Pachitariu et al., 2013; Kaifosh et al., 2014), and thus can neither distinguish functionally active cells from silent ones nor separate spatially connected but functionally different units. Some methods rely on priors about morphological properties such as size and shape patterns (Valmianski et al., 2010), and thus are very likely to fail for the astrocyte problem due to the irregular and heterogeneous morphologies of FIUs. The most well-accepted set of sophisticated tools is grounded on matrix decomposition techniques such as independent component analysis (Reidl et al., 2007; Mukamel et al., 2009), sparse dictionary learning (Diego et al., 2013), multilevel sparse matrix factorization (Andilla and Hamprecht, 2013), non-negative matrix factorization (Maruyama et al., 2014; Soelter et al., 2014), and constrained non-negative matrix factorization (Pnevmatikakis et al., 2016). However, just like CellSort, all these neuron-targeted algorithms make either an explicit or implicit assumption that pixels in an FIU are synchronized under the given imaging resolution. The violation of this assumption in astrocyte data leads both to false splits of a single FIU and to inaccurate estimates of characteristic curves of FIUs. Being blind source separation strategies, they also have difficulties in pre-determining the number of sources and in interpreting resultant overlapped components. Additionally, many of these methods make assumptions of spatial sparsity, temporal sparsity or mutual independence of Ca^2+^ signals, which are often not satisfied by astrocyte data. Last but not the least, matrix decomposition methods basically treat pixels as independent, leaving out the information embedded in spatial relationships among neighboring pixels.

### General applicability and limitations

Though designed for time-lapse imaging data of astrocytic Ca^2+^ dynamics, FASP is also potentially applicable to many other types of imaging data that have similar properties, which can be captured by the proposed model (1). Many of FASP's advantages still hold in such cases, including flexibility, outstanding performance on large-scale data, and probabilistically controlled parameter tuning.

Particularly, it is interesting to see how FASP can be applied to model neuronal Ca^2+^ dynamics (Mukamel et al., 2009). Recent advances in high-throughput time-lapse Ca^2+^ imaging of large populations of neurons (Ahrens et al., 2013; Prevedel et al., 2014) have generated a tremendous amount of data and hold the potential to understand the neuronal ensemble activities and coding. There are two possible routes to apply FASP to neuronal data. (1) When the Ca^2+^ imaging is focused on soma and hence the analysis is of cellular level, the somatic intracellular propagation of Ca^2+^ transients is so fast that the signals can be regarded as synchronized among pixels in a single neuron. So, we do not need to explicitly model the signal propagation within cell. However, the simplified version of FASP with an intracellular synchronization assumption can still help us to automate the analysis, ease the parameter setting and reduce false positive discoveries, due to the machine-learning and probabilistic nature of the FASP framework. (2) When the Ca^2+^ imaging is focused on dendrites, the neuronal Ca^2+^ signals look more like the ones in astrocytes than the somatic signals, because of the existence of calcium compartmentalization and signal propagation in dendrites (Yuste et al., 2000; Higley and Sabatini, 2008). In this case, we expect the application of FASP will be especially useful to extract information that is otherwise missed, because of its data-driven and unbiased nature. However, caution needs to be exercised. Dendritic calcium signaling may be very different from astrocytic calcium signaling, such as the frequency of events, the size and the density of regions of interest, and the signal-to-noise ratio.

More broadly, although FASP was motivated by a specific biological problem, some of the technical innovations that we developed during the process are quite generic and can be applied to other problems. For example, the order-statistics-based binarization is one of the key innovations in this paper. Due to the prevalence of segmentation problem in imaging analysis, we expect this new segmentation method may find broad applications, especially when the pixel values can be assigned statistical meaning.

One possible limitation of FASP arises from its assumption of fixed spatial positions of FIUs. Cells sometimes migrate in the field of view, and their shapes sometimes change as well. This problem can be entirely or partially solved by either pre-processing, which registers cells, or post-processing, which eliminates abnormal output FIUs.

Another concern may be about the assumption that a single pixel is associated with at most one FIU. Due to limited resolution along the z-axis or other reasons, the signals at some pixels are actually mixtures of multiple biological signals. However, without ground truth, results of blind decomposition are often difficult to explain, or turn out to be wrong in simulation experiments. Instead, FASP does not make an attempt to decompose the mixed underlying biological signals but sticks to the objective presentation of observed signal patterns. On the basis of FASP's outputs, downstream analysis can still be performed to unmix the true sources.

## Conclusions

We developed a data-driven and probabilistically principled algorithm to automatically quantify the functional status of astrocytes from astrocyte time-lapse Ca^2+^ imaging data. Integrated with a series of statistical machine learning techniques, FASP was demonstrated to be able to successfully decode complex spatiotemporal patterns of calcium signaling and control the false positive rate. We expect that broad applications of this tool would greatly facilitate analyzing astrocyte function to uncover its complicated roles in neuronal circuits.

## Ethics statement

This study was carried out in allegiance with active biological use authorization and animal protocols. The protocols were approved by institutional animal care and use committee at University of California, Davis.

## Author contributions

GY and LT conceived, designed, and coordinated the study. YXW and GY designed the mathematical modeling and executed the programming. YXW performed all the computational experiments. GS performed all the wet-lab experiments. YZW and CW helped with the algorithm design and computational experiments. YXW and GY wrote the manuscript with critical inputs from DM, GB, YW and LT.

### Conflict of interest statement

The authors declare that the research was conducted in the absence of any commercial or financial relationships that could be construed as a potential conflict of interest.
