# Supplementary material for: Automated Functional Analysis of Astrocytes from Chronic Time-Lapse Calcium Imaging Data
Source: Front Neuroinform. 2017 Jul 14;11:48. doi: 10.3389/fninf.2017.00048 (PMC5509822; doi:10.3389/fninf.2017.00048)
Supplement: Supplementary file 8 [file Presentation1.pdf]

Supplementary Material

Automated Functional Analysis of Astrocytes from Chronic Time-Lapse Calcium Imaging Data

Yinxue Wang†, Guilai Shi†, David J. Miller, Yizhi Wang, Congchao Wang, Gerard Broussard, Yue Wang, Lin Tian*, Guoqiang Yu*

† Co-first author

*** Correspondence:** Guoqiang Yu (yug@vt.edu), Lin Tian (lintian@ucdavis.edu)

1 Supplementary Videos

Supplementary Video 1, related to Figure 1 and Figure 6. Representative example of time-lapse Ca^{2+} imaging data of *in vitro* human astrocyte induced pluripotent stem cells (hiPSCs). hiPSCs were infected with lentivirus expressing EF1a-GCaMP6, and imaged under a confocal microscope three days after infection. This sample has typical properties of astrocyte Ca^{2+} imaging data: irregular and heterogeneous morphological features, spatial propagation of intracellular Ca^{2+} events with complex speed and direction patterns, and various SNRs in the same field of view. Left: the input time-lapse imaging data shown in green pseudo-color; spatial dimension 512×512; 100 frames. Right: analytical results by FASP, with FIUs labeled using carmine contours on the input data. The video of Figure 6D1. The corresponding original TIFF image stacks of all supplemental videos can be found at github.com/VTcbil/FASP as demo data for FASP's ImageJ/Fiji plugin.

Supplementary Video 2, related to Figure 4. Representative example of synthetic time-lapse Ca^{2+} imaging data. One example of synthetic data mimicking time-lapse astrocyte Ca^{2+} imaging data. Critical properties of real-world astrocyte Ca^{2+} time-lapse imaging data were taken into account to synthesize such each sample: spatial distribution and morphology of FIUs and Ca^{2+} inactive cells/units, weak edges of cells, characteristic temporal dynamics, Ca^{2+} propagation pattern, the gradually fading activities at the boundaries of FIUs and heterogeneous SNRs. Left: the input time-lapse imaging data shown in green pseudo-color; spatial dimension 256×256; 100 frames. Right: analytical results by FASP, with FIUs labeled using carmine contours on the input data.

Supplementary Video 3, related to Figure 6. Slow propagation of Ca^{2+} elevations constitutes fundamental problems for CellSort in detecting astrocytic FIUs. CellSort's analytical results on the same hiPSCs data sample as in Supplementary Video 1. Left: the input time-lapse imaging data shown in green pseudo-color; spatial dimension 512×512; 100 frames. Right: analytical results by CellSort, with regions of interest (ROIs) detected by CellSort highlighted using contours on the input

data. Different ROIs were labelled using different colors to show the overlap between ROIs. The video of Figure 6D2.

Supplementary Video 4, related to Figure 7. FASP captured the association between ATP dose and the number of responding FIUs: a control sample with no ATP. The video of the upper left figure in Figure 6E. FASP was applied to the Ca^{2+} time-lapse images of rat hippocampal astrocytes, monitoring their calcium dynamics as responses to quantitatively controlled ATP stimulation. The control sample only contained one FIU with spontaneous activity. Left: original input imaging data of a control sample with no ATP was added in; spatial dimension 512×512; 100 frames. Right: analytical results by FASP, with FIUs labeled using carmine contours on the input data.

Supplementary Video 5, related to Figure 7. FASP captured the association between ATP dose and the number of responding FIUs: a sample with 1uM ATP applied. The video of the upper right figure in Figure 6E. A sample of rat hippocampal astrocytes, with 1uM ATP applied. Panels similar as in Supplementary Video 4.

Supplementary Video 6, related to Figure 7. FASP captured the association between ATP dose and the number of responding FIUs: a sample with 10uM ATP applied. The video of the bottom left figure in Figure 6E. A sample of rat hippocampal astrocytes, with 10uM ATP applied. Panels similar as in Supplementary Video 4.

Supplementary Video 7, related to Figure 7. FASP captured the association between ATP dose and the number of responding FIUs: a sample with 100uM ATP applied. The video of the bottom right figure in Figure 6E. A sample of rat hippocampal astrocytes, with 100uM ATP applied. Panels similar as in Supplementary Video 4.

2 Supplementary Toolbox User Manual

2.1 Installation

Download file “FASP_.jar” from https://github.com/VTcbil/FASP. To install the FASP plugin, simply save or copy it into the folder “\Fiji.app\plugins\”, then click “Help ▸ Refresh Menus” or restart Fiji/ImageJ. The FASP plugin will become available in Fiji/ImageJ's “Plugins” menu.

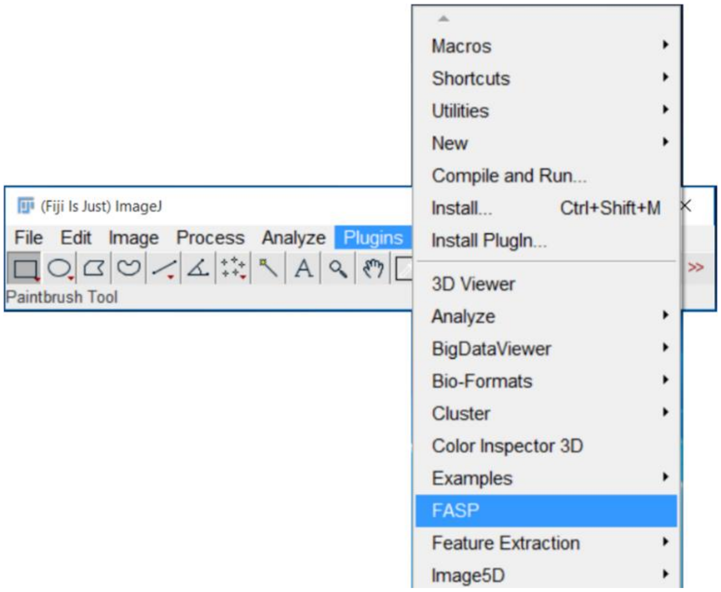

The plugin has been tested and successfully run on ImageJ version ≥ 1.49q. If users encounter problems using older versions of ImageJ, please update your ImageJ to a newer version ≥ 1.49q.

2.2 Input

FASP is designed for single-channel gray-scale time lapse image stack. So far we only support 8-bit or 16-bit images of any file format supported by Fiji/ImageJ. Open the image stack of interest in Fiji/ImageJ.

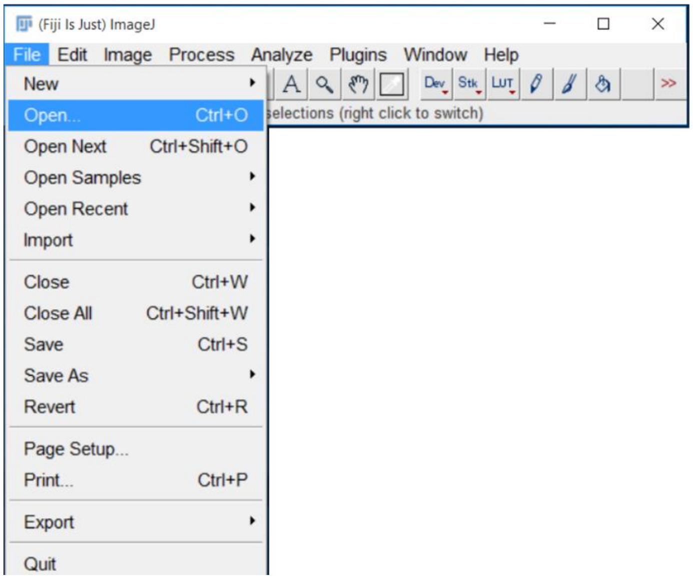

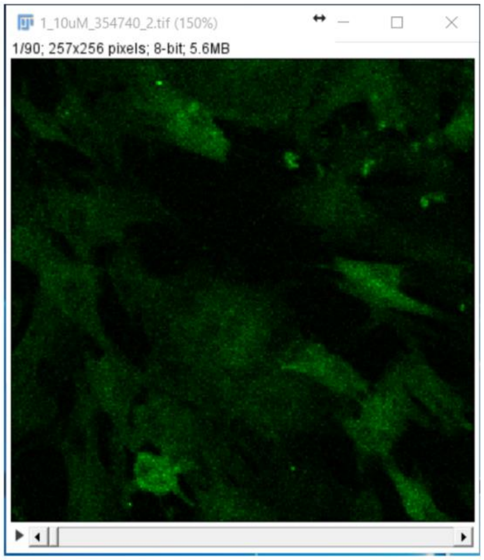

(**Note 1:** Fiji/ImageJ typically provides users with “Lookup Tables” under “Image” menu that can be applied to single-channel grayscale images or image stacks to produce pseudo-color images. Some colors may make manual proofreading easier, such as green color. FASP can be applied to single-channel grayscale image stacks of any pseudo color, and the results will be shown as overlay on the input pseudo-color image stack.)

(**Note 2:** FASP works on single-channel image stacks. If the input image stack contains more than one channel, FASP will report an alert and exit automatically. Users can resort to other tools, such as Fiji/ImageJ function “Split Channels” under “Image\Color”, to make sure the input data for FASP is single-channel.)

2.3 Parameter Setting

With the input image stack loaded, run FASP from the plugins menu. A dialogue window will occur, allowing users to adjust the input parameters. Suggested values of parameters are given as default in the textboxes. After the parameter setting is done, click “OK”.

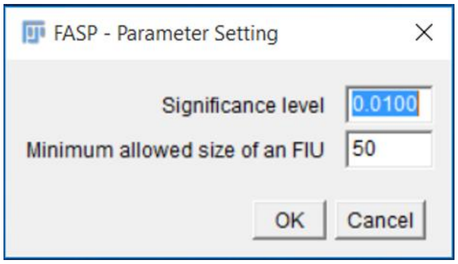

2.4 Processing Procedure

Since FASP is a completely automated algorithm, NO interactive user input is needed while FASP is running. The progress of the program will be shown using a progress bar in the bottom right corner of Fiji/ImageJ toolbar. Besides computing platform, the running time of FASP also depends on the activity level of the imaged cells, the spatiotemporal scales of input image stack, and the minimum allowed size of FIUs.

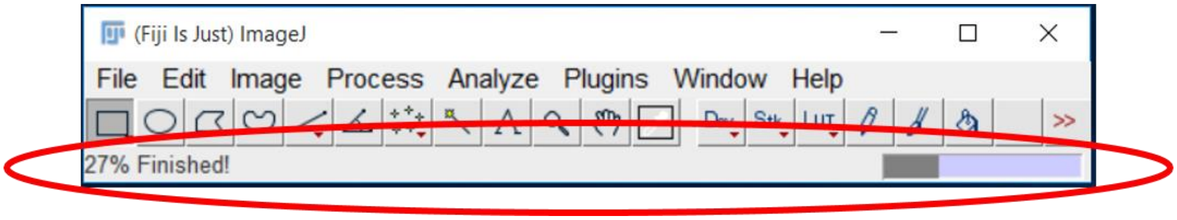

2.5 Outputs

The results are visualized using tables and the ROI manager of Fiji/ImageJ. All FIUs are circled and labeled on the original image stack. The edges of FIUs are indicated as yellow, and a label is put on each FIU to show the ID of this FIU. If the yellow edges disappear due to some reason/operation, users may reset the edge overlay by checking the checkbox “show all” on the bottom right.

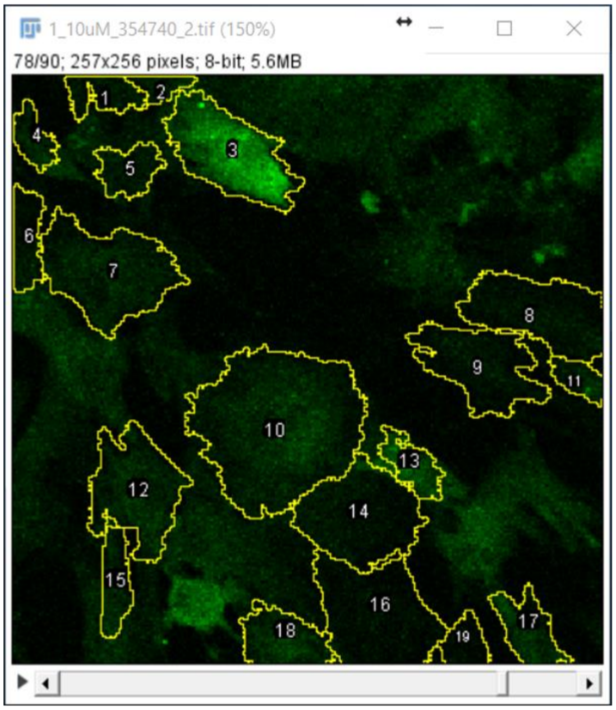

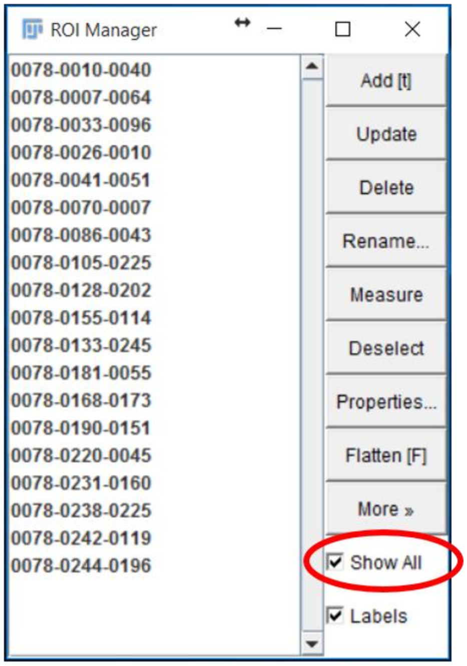

If a user is particularly interested in one FIU, she/he can click on the label of the FIU of interest to inspect its characteristic curve (in $\Delta F/F_0$). The selected FIU will be highlighted with blue edges on the image stack.

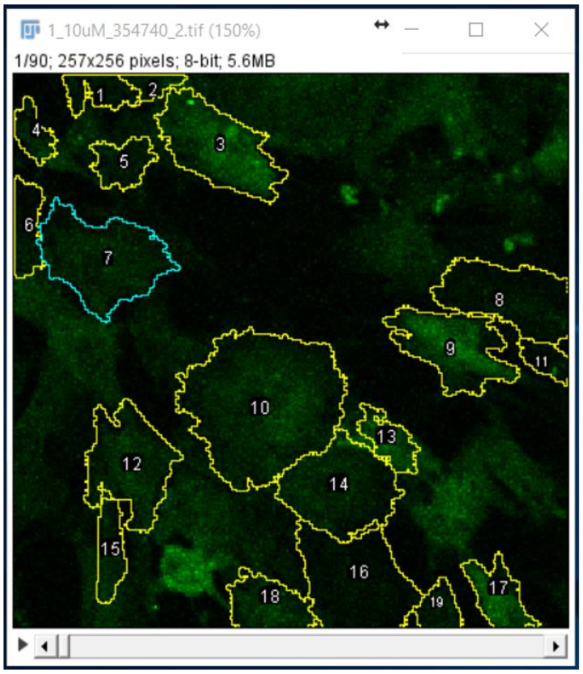

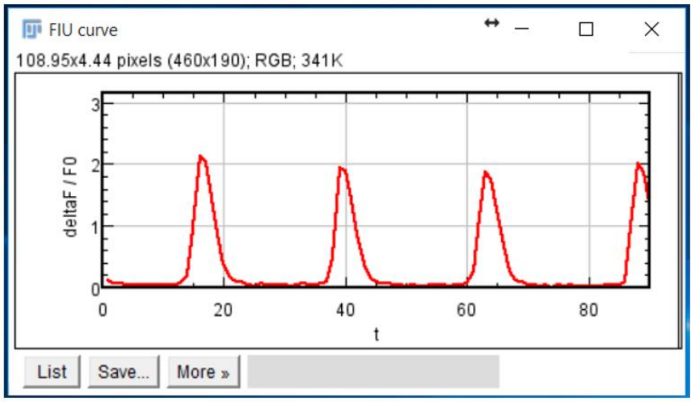

Besides, to make it easier for users to save the results, a table is also given containing the characteristic curves (in $\Delta F/F_0$) of all FIUs.

Characteristic Curve Table

File Edit Font

Label	t1	t2	t3	t4	t5	t6	t7	t8	t9	t10	t11	t12	t13	t14	t15	t16
FIU1	0.073	0.087	0.069	0.083	0.083	0.089	0.048	0.832	0.959	0.799	0.468	0.308	0.108	0.115	0.045	0.04[illegible]
FIU2	0.073	0.134	0.071	0.070	0.010	0.104	0.100	0.004	0.107	0.122	0.144	0.086	0.023	0.109	0.083	0.11[illegible]
FIU3	0.039	0.033	0.033	0.022	0.032	0.027	0.023	0.026	0.051	0.141	0.805	1.597	1.619	1.243	0.742	0.33[illegible]
FIU4	0.036	0.062	0.062	0.073	0.043	0.048	0.041	0.045	0.019	0.033	0.059	0.094	0.100	0.132	0.063	0.04[illegible]
FIU5	0.045	0.037	0.071	0.036	0.087	0.007	0.044	0.116	0.112	0.070	0.041	0.102	0.085	0.091	0.065	0.08[illegible]
FIU6	0.058	0.095	0.054	0.112	0.169	0.980	1.077	0.785	0.592	0.332	0.229	0.098	0.134	0.101	0.063	0.05[illegible]
FIU7	0.113	0.070	0.063	0.057	0.054	0.042	0.041	0.051	0.049	0.045	0.053	0.042	0.085	0.182	0.962	2.13[illegible]
FIU8	0.052	0.051	0.046	0.050	0.055	0.026	0.049	0.019	0.050	0.039	0.038	0.033	0.053	0.072	0.038	0.05[illegible]
FIU9	1.989	1.166	0.569	0.211	0.121	0.086	0.069	0.068	0.054	0.067	0.053	0.060	0.066	0.146	1.776	2.60[illegible]

The basic information of each FIU is listed in a table, including its area (in pixel) and the magnitude (max $\Delta F/F_0$) of this characteristic curve.

Feature Table

File Edit Font

Label	area(pixel)	max DeltaF/F0
FIU1	436	0.975
FIU2	225	1.346
FIU3	1678	1.699
FIU4	340	1.690
FIU5	472	0.654
FIU6	511	1.077
FIU7	1966	2.134
FIU8	1592	2.182
FIU9	1429	2.608
FIU10	4031	1.494
FIU11	299	1.587
FIU12	1578	3.154
FIU13	447	0.557
FIU14	1816	1.337
FIU15	493	2.101
FIU16	1962	2.316
FIU17	600	2.017
FIU18	766	1.250
FIU19	337	1.610

And some overall assessment of the sample is also provided in a summary table.

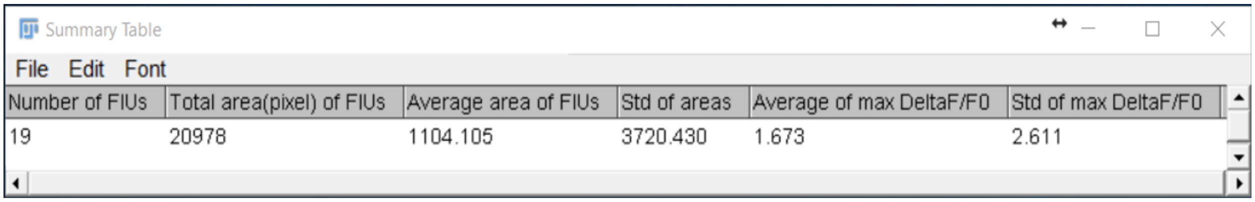

Number of FIUs	Total area(pixel) of FIUs	Average area of FIUs	Std of areas	Average of max DeltaF/F0	Std of max DeltaF/F0
19	20978	1104.105	3720.430	1.673	2.611

To save the results, the user can use the export/save function of Fiji/ImageJ for ROI manager, images and tables.

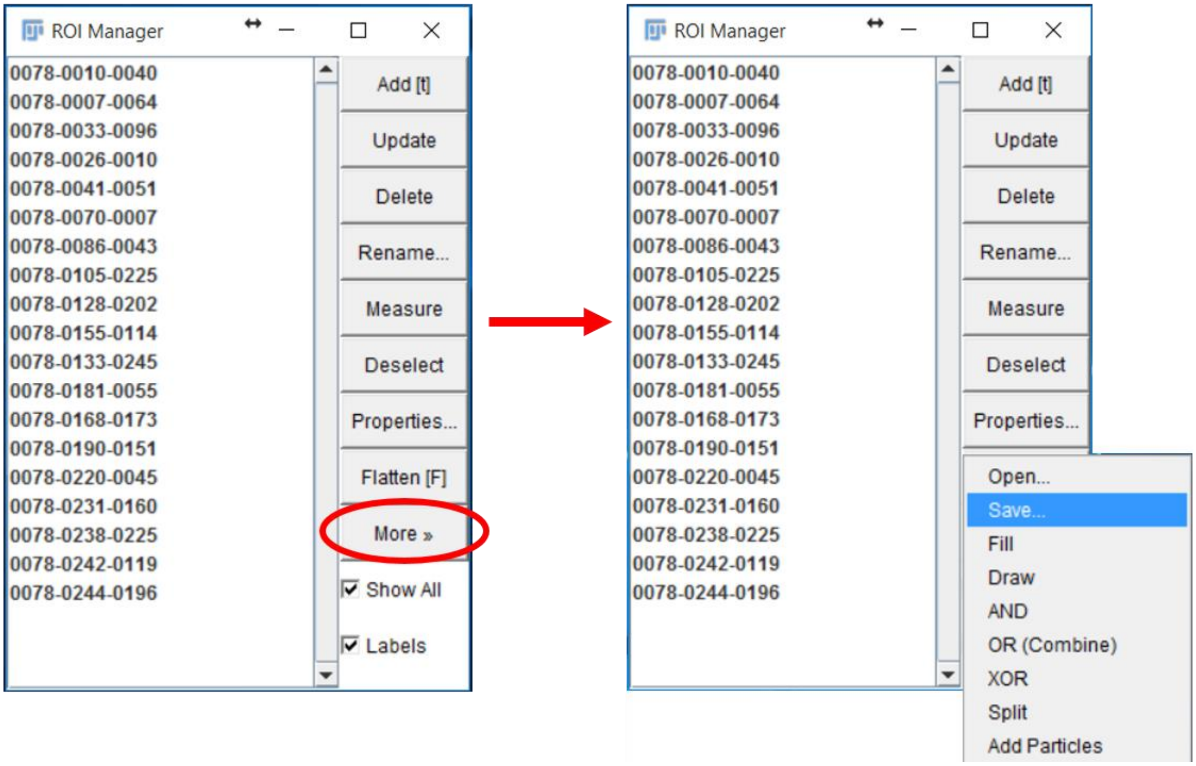

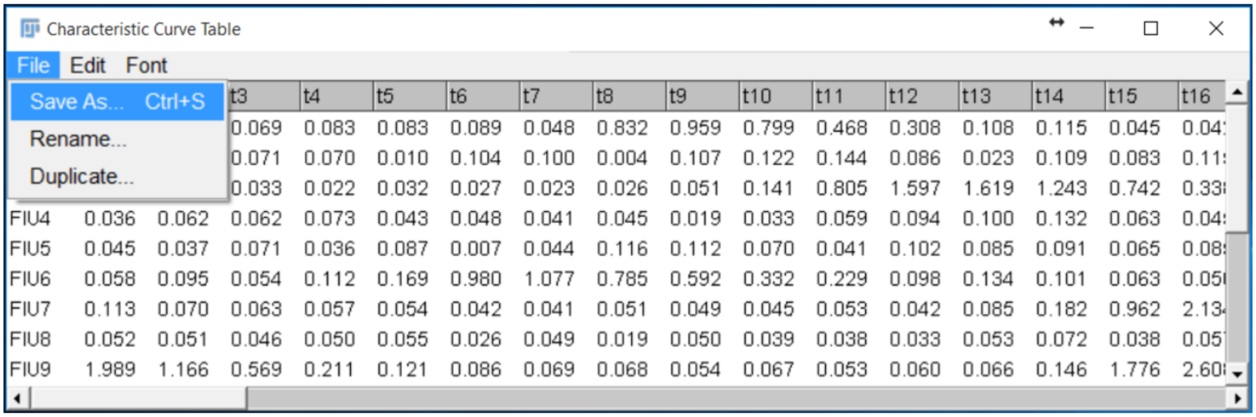

[illegible]	[illegible]	[illegible]	t3	t4	t5	t6	t7	t8	t9	t10	t11	t12	t13	t14	t15	t16
[illegible]	[illegible]	[illegible]	0.069	0.083	0.083	0.089	0.048	0.832	0.959	0.799	0.468	0.308	0.108	0.115	0.045	0.04[illegible]
[illegible]	[illegible]	[illegible]	0.071	0.070	0.010	0.104	0.100	0.004	0.107	0.122	0.144	0.086	0.023	0.109	0.083	0.11[illegible]
[illegible]	[illegible]	[illegible]	0.033	0.022	0.032	0.027	0.023	0.026	0.051	0.141	0.805	1.597	1.619	1.243	0.742	0.33[illegible]
FIU4	0.036	0.062	0.062	0.073	0.043	0.048	0.041	0.045	0.019	0.033	0.059	0.094	0.100	0.132	0.063	0.04[illegible]
FIU5	0.045	0.037	0.071	0.036	0.087	0.007	0.044	0.116	0.112	0.070	0.041	0.102	0.085	0.091	0.065	0.08[illegible]
FIU6	0.058	0.095	0.054	0.112	0.169	0.980	1.077	0.785	0.592	0.332	0.229	0.098	0.134	0.101	0.063	0.05[illegible]
FIU7	0.113	0.070	0.063	0.057	0.054	0.042	0.041	0.051	0.049	0.045	0.053	0.042	0.085	0.182	0.962	2.13[illegible]
FIU8	0.052	0.051	0.046	0.050	0.055	0.026	0.049	0.019	0.050	0.039	0.038	0.033	0.053	0.072	0.038	0.05[illegible]
FIU9	1.989	1.166	0.569	0.211	0.121	0.086	0.069	0.068	0.054	0.067	0.053	0.060	0.066	0.146	1.776	2.60[illegible]
